# Benzenesulfonamide derivatives as *Vibrio cholerae* carbonic anhydrases inhibitors: a computational-aided insight in the structural rigidity-activity relationships

**DOI:** 10.1080/14756366.2023.2201402

**Published:** 2023-04-19

**Authors:** Marialuigia Fantacuzzi, Ilaria D’Agostino, Simone Carradori, Francesco Liguori, Fabrizio Carta, Mariangela Agamennone, Andrea Angeli, Filomena Sannio, Jean-Denis Docquier, Clemente Capasso, Claudiu T. Supuran

**Affiliations:** a Department of Pharmacy, “G. d’Annunzio” University of Chieti-Pescara, Chieti, Italy; b Neurofarba Department, Section of Pharmaceutical and Nutraceutical Sciences, University of Florence, Florence, Italy; c Department of Medical Biotechnologies, University of Siena, Siena, Italy; d InBioS, Centre for Protein Engineering, University of Liège, Liège, Belgium; e Department of Biology, Agriculture and Food Sciences, National Research Council (CNR), Institute of Biosciences and Bioresources, Naples, Italy

**Keywords:** Carbonic anhydrase, sulfonamides, *Vibrio cholerae*, imidazolidinone, isoform selectivity, homology modelling, docking, MD

## Abstract

*Vibrio cholerae* causes life-threatening infections in low-income countries due to the rise of antibacterial resistance. Innovative pharmacological targets have been investigated and carbonic anhydrases (CAs, EC: 4.2.1.1) encoded by *V. cholerae* (*Vch*CAs) emerged as a valuable option. Recently, we developed a large library of *para*- and *meta*-benzenesulfonamides characterised by moieties with a different flexibility degree as CAs inhibitors. Stopped flow-based enzymatic assays showed strong inhibition of *Vch*αCA for this library, while lower affinity was detected against the other isoforms. In particular, cyclic urea **9c** emerged for a nanomolar inhibition of *Vch*αCA (*K_I_* = 4.7 nM) and high selectivity with respect to human isoenzymes (SI≥ 90). Computational studies revealed the influence of moiety flexibility on inhibitory activity and isoform selectivity and allowed accurate SARs. However, although *Vch*CAs are involved in the bacterium virulence and not in its survival, we evaluated the antibacterial activity of such compounds, resulting in no direct activity.

## Graphical Abstract

**Figure:**
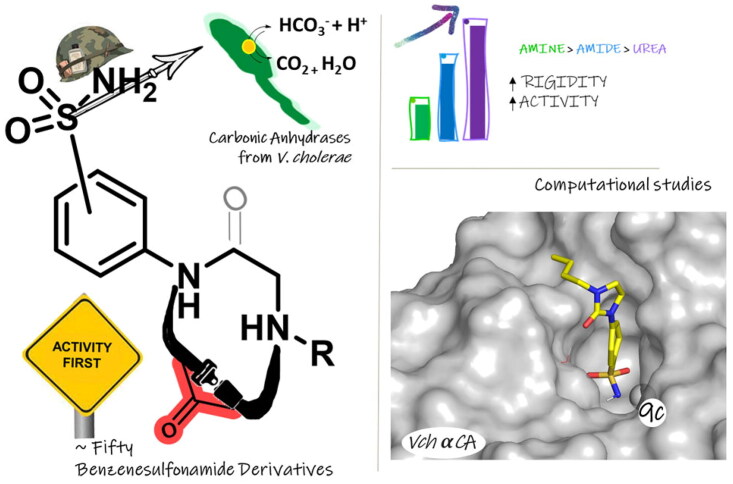

## Introduction

The COVID-19 pandemic represented an unanticipated global issue, responsible for over 6.8 M deaths worldwide and a big concern in Public Healthcare in relation to the impairment of human immune defences, thus unable to fight secondary microbial infections that became potentially fatal^1^. However, besides these direct and economic burdens, the outbreak of COVID-19 was also responsible for the crowding of hospitals and the consequent slowing down of other disease treatments and surgery, such as those related to cancer patients^2^. Moreover, underdeveloped countries significantly suffered from the COVID-19 disruption of humanitarian aid programs, fundamental in the life-saving safe water, sanitation, and hygiene (WASH) techniques and the diagnosis, treatment, and vaccination campaigns for those diseases, such as cholera, that are a passed threat for industrialised countries^3^^,^^4^.

Cholera, also referred to as “blue death”, is an acute diarrheal illness that became fatal if left untreated (50–70% mortality rate) and provoked seven pandemics till the 19th century and a consequent number amounting to an impressive 143 000 deaths per year^5^. In recent years, its incidence is most evident in those areas with low hygienic conditions and limited access to safe drinking water and food, in particular after cataclysmic events or during armed conflicts, such as sub-Saharan African countries, *i.e.* Niger, Nigeria, Ethiopia, and Sudan, India, and Bangladesh^6–8^. The alarming data from these areas envisaged the institutions of a worldwide strategy to control the disease named “Ending Cholera: a global roadmap to 2030” in 2017 aimed at reducing mortality by 90%. However, a significative increase in reported cholera cases was noticed during the COVID-19 pandemic compared to previous years^7^.

Cholera aetiology is related to the infection by the Gram-negative curved rod-shaped *Vibrio cholerae,* but only two serogroups, O1 and O139, can cause illness^9^, characterised by a massive loss of water and electrolytes, resulting in severe dehydration and hypovolemic shock. Thus, the pharmacological therapies are usually based on intravenous rehydration and the administration of antibiotics, such as a single dose of doxycycline as first-line treatment, erythromycin, azithromycin, norfloxacin, ciprofloxacin, and trimethoprim-sulfamethoxazole combination^10^. However, several cases of bacterial resistance were reported^11–14^ and make the search for new antibiotics and their development urgently needed, although some oral vaccines (the FDA-licensed Vaxchora® and the WHO-prequalified Dukoral^®^, ShanChol^®^, and Euvichol-Plus®/Euvichol®) are already in clinical use.

In the last decades, carbonic anhydrases (CAs, E.C. 4.2.1.1) emerged for their multifaceted physio-pathological roles and, thus, their high potential as pharmacological targets in medicinal chemistry and the antibacterial field^15^. CAs are ubiquitous metalloenzymes involved in the physiological balance between carbonic dioxide and bicarbonate anion in the cell, thus governing the pH homeostasis, the secretion of electrolytes, the carboxylation reactions, and other metabolic pathways^16^^,^^17^. Herein, the interest in inhibiting CAs with antibacterial purposes is amply justified and recently confirmed in *in vivo* studies^18–22^.

The genome of *V. cholerae* species encodes for three CAs, belonging to three of the eight different classes of CA isoforms: the α-, also present in mammalians, and the β- and the γ-isoenzymes^23^^,^^24^. Although the role of *Vch*CAs has not been well established yet, accumulating evidence relates their function to the etiopathogenesis of the *V. cholerae* infection^25^. After the invasion of the host and the colonisation of the upper small intestine epithelial layer, the pathogen penetrates the mucus and attaches to the microvilli via the bacterial pili, where it releases its endotoxin (cholera toxin, CT), the main virulence factor, that triggers the increased secretion of water, sodium and potassium cations and bicarbonate anion into the lumen of the intestine, leading to severe dehydration^26^. The small intestine maintains an alkaline pH environment through the pancreatic release of sodium bicarbonate, an inducer of the CT expression^25^. However, the absence of genes encoding for bicarbonate transporters in *V. cholerae* lets us hypothesise the use of CAs as a bicarbonate-assimilating system in the cell.

Moreover, as assessed through the STRING web tool (https://string-db.org/ accessed on March 4th, 2022)^27^, *Vch*CAs participate in several functional and physical protein-protein association networks, such as proteins belonging to the sulfate permease family, those involved in the degradation of long-chain fatty acids, fumarate hydratase, uridine kinase, etc. Relevantly, *Vch*CAs also seem to be associated with the thioredoxin system, fundamental in the physiology and pathogenesis of bacteria due to its influence on the expression of many genes, in the reduction of cytoplasmic proteins and hydrogen peroxide and, in general, cell division, energy transduction, oxidative stress response, transcriptional regulation, phage assembly and propagation^28^.

After the isolation and characterisation of the three isoenzymes from *V. cholerae*^29^^,^^30^, several studies have been reported on the development of potent inhibitors, most of them selective, endowed with different chemical scaffolds^31^, such as benzoxaboroles^32^, indole-based hydrazones^33^, sulfamides (-NHSO_2_NH_2_)^34^, *N-*hydroxy-ureas^35^, and differently substituted benzenesulfonamides designed through the tail approach^36–39^.

**Figure 1.**
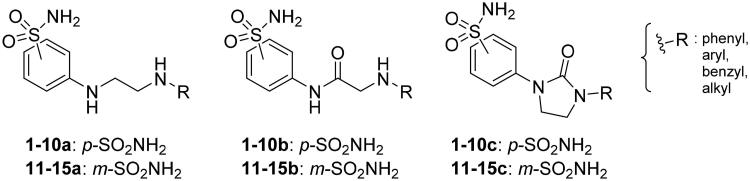
Compounds investigated in this work.

We recently developed a series of benzenesulfonamides as antitumor agents, highlighting, in some cases, a clear trend in the inhibitory profile according to the presence of specific moieties able to confer different rigidity to the chemical structure (Figure 1)^40^^,^^41^.

However, the compounds showed no higher selectivity towards the enzymes of interest, CA IX and CA XII, with respect to the cytosolic isoforms I and II. Thus, we investigated the possible inhibition of one or more different CAs, such as those from the bacterial *V. cholerae.*

## Results and discussion

### Rationale and preparation of the library

**Figure 2.**
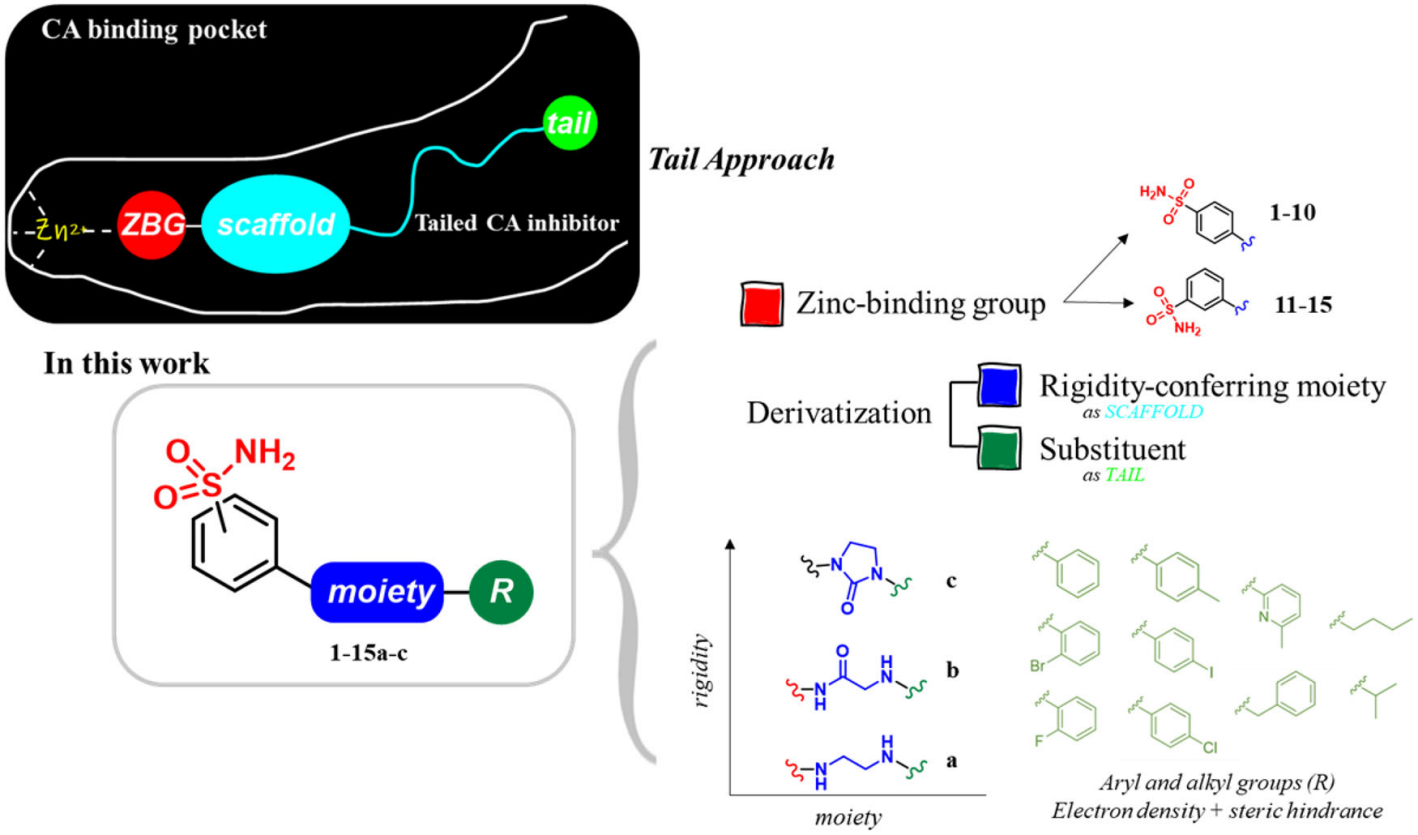
Overview of the tail approach and the design strategy used in the current work.

The derivatives design was inspired by *the tail approach*, a MedChem strategy that has been applied for the first time in the CA inhibition field in 1999^42^^,^^43^. In the beginning, it was aimed to enhance the pharmacokinetic properties of such compounds^44^, then, it was largely employed to address the isoform selectivity issue. Generally, tailed CA inhibitors are composed of three elements: a zinc-binding group (ZBG, in red, Figure 2), a main scaffold with a spacer (in cyan, Figure 2), and the hydrophobic tail (in light green, Figure 2).

This strategy is based on the idea to allow the compound zinc-binding group to reach the enzyme metal in the binding cavity and to establish a complex and well-defined interaction network with the isoform-specific enzyme residues (most of which are hydrophobic) in the entry of the binding site through the scaffold and tail portions.

In the current work, the design of derivatives **1–15a–c** involved the use of 4- and 3-benzenesulfonamides as zinc-binding groups (in red, Figure 2) and different derivatization elements as binary systems composed of flexibility/rigidity-conferring moiety (in blue, Figure 2), such as the amine (**a**), the amide (**b**), and the cyclic urea (**c**) function (in increasing order of rigidity in Figure 2), in lieu of the traditional scaffold-spacer component, and a specific aryl or alkyl substituent (R, in green, Figure 2) endowed with different electron density and steric hindrance properties as the compound tail.

Aimed at exploring the enzymatic inhibitory profile of the library of compounds **1–15a–c** and discovering promising activities for further development as antibacterial agents, we evaluated their ability to inhibit the three CAs from the pathogen *V. cholerae*. Compounds **1–15a–c** were prepared as reported^40^.

### In vitro inhibition of VchCAs and preliminary SAR considerations

The inhibition profiles for sulfonamides **1–15a**–**c** and the reference **AAZ** against α-, β-, and γ-CAs from *V. cholerae* were determined through the stopped-flow CO_2_ hydrase assay^45^, and as a comparison, inhibitory data on the physiologically relevant *h*CAs I and II are reported as inhibition constants (*K_I_*s) (Table 1).

**Table 1.** Inhibition data of sulfonamides **1–15a–c** and reference compound **AAZ** on *h*CAs I and II and *Vch*CAs through the stopped-flow CO_2_ hydrase assay. 
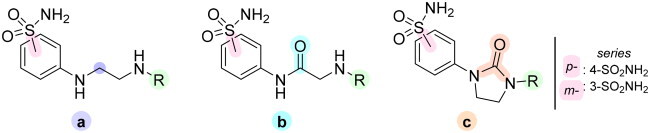

Cpd	Scaffold	Series	*R*	*K*_I_ (nM)					SI [$\frac{KI\;\mathrm{hCA}\;I}{KI\;\mathrm{Vch}\alpha \mathrm{CA}}$]	SI [$\frac{KI\;\mathrm{hCA}\;\mathrm{II}}{KI\;\mathrm{Vch}\alpha \mathrm{CA}}$]
				*h*CA I^a^	*h*CA II^a^	*Vch*αCA	*Vch*βCA	*Vch*γCA		
**1**	**a**	*p-*	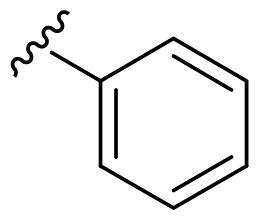	25.4	7.2	78.1	18 300	n.a.	0.3	0.1
**2**	**a**	*p-*	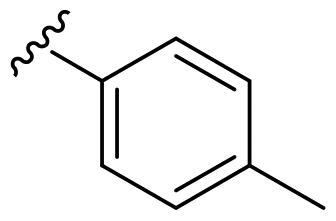	9.7	3.1	43.1	16 800	39 900	0.2	0.1
**3**	**a**	*p-*	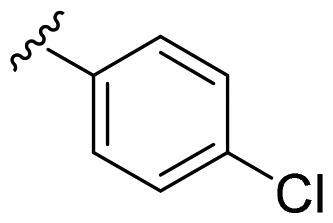	39.0	47.0	27.4	53 800	n.a.	1.4	1.7
**4**	**a**	*p-*	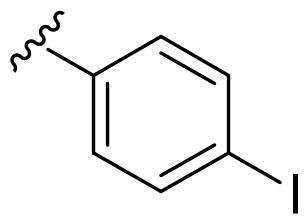	787	646	65.0	136 400	n.a.	12.1	9.9
**5**	**a**	*p-*	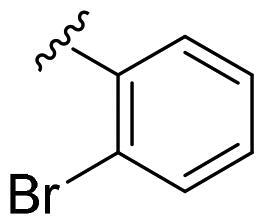	939	908	64.1	89 200	n.a.	14.7	14.2
**6**	**a**	*p-*	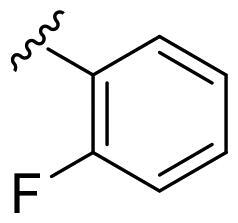	75.3	8.2	36.2	100 800	73 700	2.1	0.2
**7**	**a**	*p-*	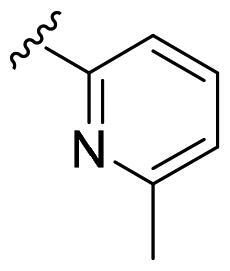	87.0	31.4	37.0	19 000	69 800	2.4	0.9
**8**	**a**	*p-*	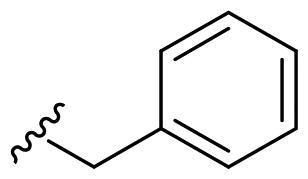	169	32.7	23.0	12 400	52 700	7.4	1.4
**9**	**a**	*p-*	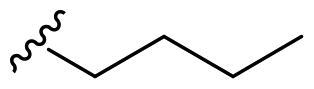	538	82.2	78.8	115 000	n.a.	6.8	1.0
**10**	**a**	*p-*	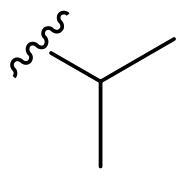	896	338	7.5	12 500	92 900	119.5	45.0
**11**	**a**	*m-*	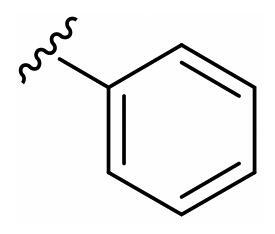	7.0	4.1	63.0	15 600	n.a.	0.1	0.1
**12**	**a**	*m-*	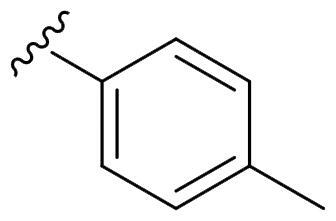	>1000	901	44.1	173 200	79 000	>22.7	20.4
**13**	**a**	*m-*	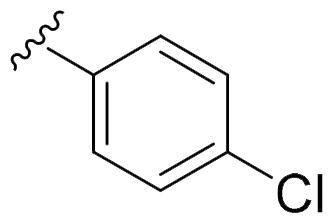	346	15.7	75.6	59 200	5600	4.6	0.2
**14**	**a**	*m-*	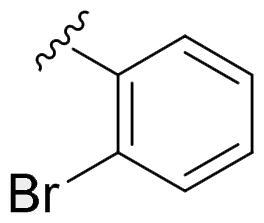	7.6	3.3	63.5	97 000	n.a.	0.1	0.1
**15**	**a**	*m-*	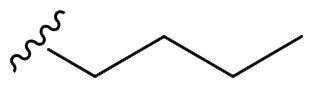	151	43.2	80.3	56 500	69 300	1.9	0.5
**1**	**b**	*p-*	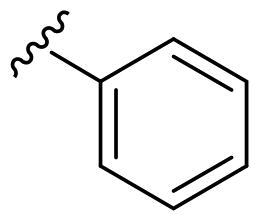	62.6	3.2	53.0	17 700	49 400	1.2	0.1
**2**	**b**	*p-*	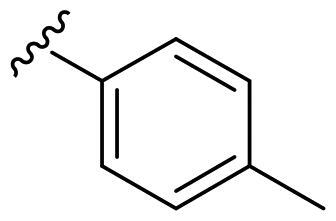	6.6	82.4	43.1	97 000	82 600	0.2	1.9
**3**	**b**	*p-*	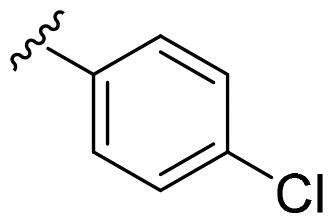	16.7	6.0	66.0	116 400	86 900	0.3	0.1
**4**	**b**	*p-*	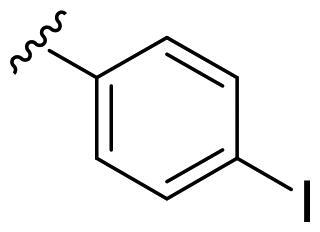	>1000	909	22.8	157 300	72 500	>43.9	39.9
**5**	**b**	*p-*	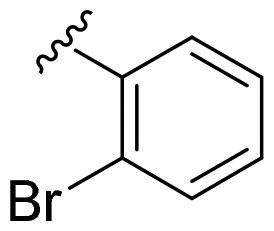	29.8	3.0	62.0	47 400	87 700	0.5	0.1
**6**	**b**	*p-*	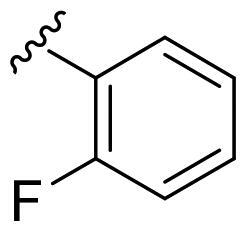	18.4	4.8	79.0	51 400	93 100	0.2	0.1
**7**	**b**	*p-*	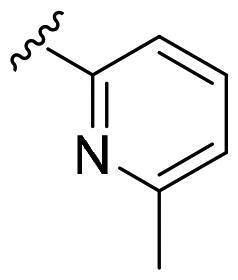	15.3	4.7	17.0	73 100	59 800	0.9	0.3
**8**	**b**	*p-*	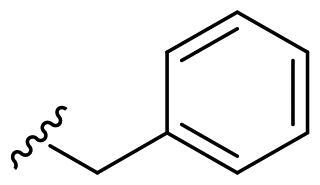	26.5	5.0	61.4	53 200	n.a.	0.4	0.1
**9**	**b**	*p-*	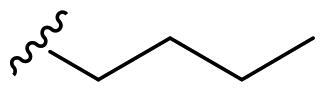	160	8.4	78.6	141 400	n.a.	2.0	0.1
**10**	**b**	*p-*	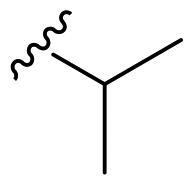	92.0	26.6	36.2	4000	58 300	2.5	0.7
**11**	**b**	*m-*	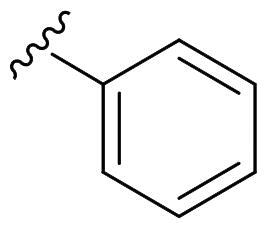	443	35.5	59.4	132 500	n.a.	7.5	0.6
**12**	**b**	*m-*	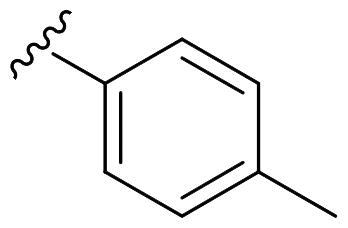	86.4	8.2	73.1	120 800	9600	1.2	0.1
**13**	**b**	*m-*	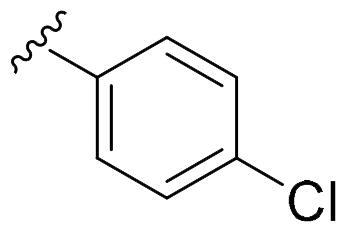	269	32.4	75.5	70 400	n.a.	3.6	0.4
**14**	**b**	*m-*	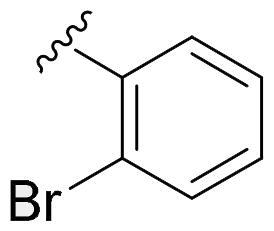	20.3	86.0	72.3	16 000	72 400	0.3	1.9
**15**	**b**	*m-*	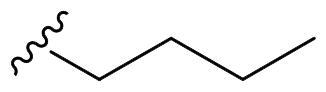	83.7	37.4	66.4	116 400	73 600	1.3	0.6
**1**	**c**	*p-*	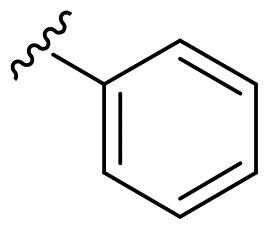	54.4	4.3	7.1	134 000	5000	7.7	0.6
**2**	**c**	*p-*	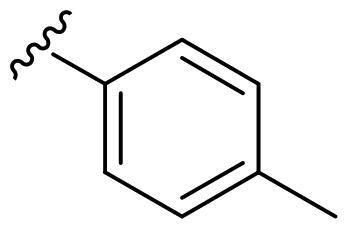	16.1	3.7	27.0	83 600	3500	0.60	0.14
**3**	**c**	*p-*	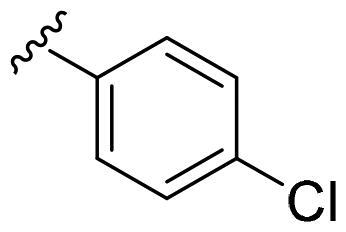	194	46.3	47.0	106 000	50 200	4.1	1.0
**4**	**c**	*p-*	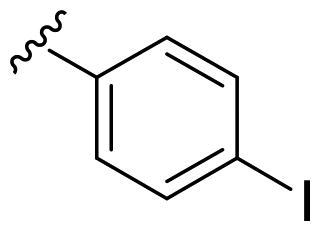	92.7	13.0	13.0	116 000	72 200	7.1	1.0
**5**	**c**	*p-*	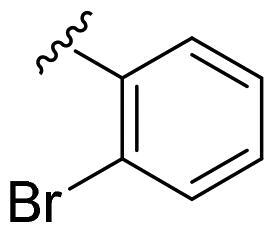	6.2	3.4	47.2	n.a.	73 200	0.1	0.1
**6**	**c**	*p-*	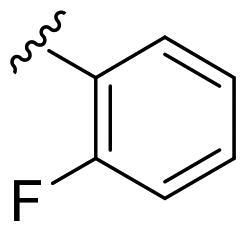	7.8	3.1	9.7	108 000	82 800	0.8	0.3
**7**	**c**	*p-*	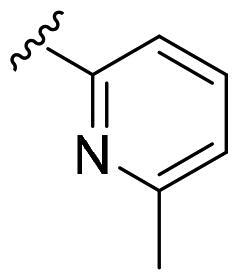	54.6	7.2	11.0	122 000	80 200	5.0	0.7
**8**	**c**	*p-*	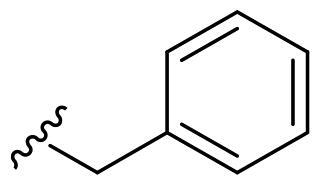	8.0	3.1	2.6	127 200	8100	3.1	1.2
**9**	**c**	*p-*	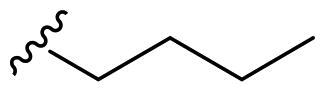	604	423	4.7	113 100	54 900	128.4	90.0
**10**	**c**	*p-*	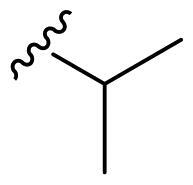	8.5	3.2	9.8	113 200	64 500	0.9	0.3
**11**	**c**	*m-*	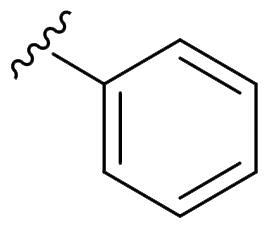	60.6	31.1	50.3	100 200	6500	1.2	0.6
**12**	**c**	*m-*	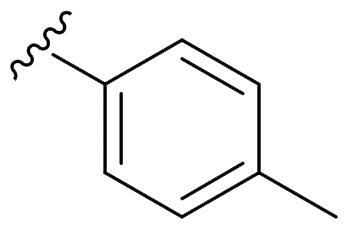	462	500	11.0	n.a.	76 000	42.0	45.5
**13**	**c**	*m-*	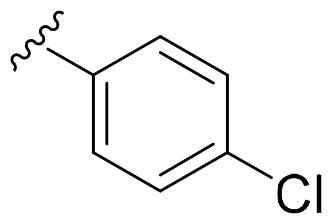	30.3	84.1	4.0	121 800	73 700	7.6	21.0
**14**	**c**	*m-*	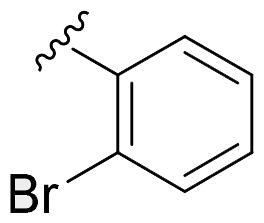	8.0	54.4	10.0	132 100	81 300	0.8	5.4
**15**	**c**	*m-*	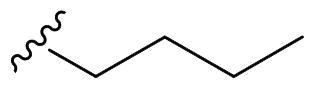	36.3	8.0	47.0	122 000	8000	0.8	0.2
**AAZ**				250	12.1	6.8	451	473	36.8	1.8

*K*_I_ values are reported as means of three independent experiments by a stopped-flow technique. Errors are in the range of ± 5–10% of the reported values. Acetazolamide (AAZ) was used as a reference control in these assays. *Vch*CAs are from *V. cholerae* species. Selectivity index (SI) values are calculated as indicated in the table. Compounds are presented based on the molecular scaffold (***a***, amine, ***b***, amide, and ***c***, urea) and the (*meta*- or *para*-) position with respect to the benzenesulfonamide core. n.a.: not active at 100 µM.

^a^Data from Ref. 40.

Based on the reported inhibition data, several structure-activity-relationship (SAR) considerations can be drawn, especially regarding inhibition spectrum and selectivity. Overall, all the compounds showed a nanomolar inhibitory activity against *h*CAs I and II^40^.

Furthermore, the assessment of the inhibitory activity on α-CA from *V. cholerae* highlights the structural differences among the binding pockets of the human and bacterial enzymes. The *in vitro* experiments seem to confirm the bigger size of the *Vch*αCA binding pocket, as outlined by the good activity of the 4-iodophenyl (**4a**) and 2-bromophenyl (**5a**) derivatives. Remarkably, pyridinyl-bearing compound **7b** results to be the most active among the amido library (scaffold **b**), while the bulky alkyl substituent of **10a** confers a higher potency to the amino *para*-series (scaffold **a**). The rigidity of scaffold **c** contributes to reducing the *K*_I_ values, resulting in notable inhibitory profiles for almost all the *para*-sulfonamides and monosubstituted phenyl derivatives **12–14c**. In general, all tested cyclic compounds were found active in inhibiting the target enzymes, even if they failed to reach the high potency of **AAZ** in some cases, especially for those endowed with scaffolds **a** and **b**.

The whole set of compounds exerts a less potent inhibitory activity on the β-CA isoform of *V. cholerae*, as shown by the *K*_I_s in the micromolar range. Low activity was found for halophenyl derivatives characterised by **a** scaffold (**4–6a** and **14a**). Also, the same trend of inhibition was observed for the acyclic derivatives, highlighting a better activity profile of the bulky **10a** than the linear derivatives **9a** and **15a**. Regarding the aromatic moieties on scaffold **b**, the unsubstituted phenyl ring positively affects the activity, conferring the best inhibitory profile among the para-series (**1b**) and the worst among the *meta*-series (**11b**). However, the most active amide is the *i*-propyl compound **10b** with a *K*_I_ value of 4.0 µM.

Finally, the tested compounds show a very low (in the micromolar range) activity on the γ isoform. In particular, in the *para*-benzenesulfonamide amine series (scaffold **a**) the inactivity of the phenyl derivative **1a** seems to be restored only in the presence of an electron donor (methyl) substituent on the ring (**2a**), while the introduction of one halogen in *para* or *ortho* position (**3–5a**) still corresponds to a lack of activity, except for the fluorophenyl compound **6a**. Not relevant *K*_I_ values were also detected on the other derivatives. Inactivity also for the phenyl derivative in the *meta-***a** series (**11a**), but this time, restored by tolyl moiety in **12a** and still improved by the chlorophenyl derivative **13a**. The butyl tail improves the *Vch*γCA inhibition in the *meta-*series (**15a**) with respect to the *para*- one (**9a**). In the **b** library, the only interesting compound, with a *K*_I_ of 9.6 µM, bears the tolyl ring (**12b**) in the *meta*-series. The cyclic ureas (scaffold **c**) show notably low micromolar activities, especially for the unsubstituted phenyl derivatives (**1c** and **11c**).

The panel of tested CAs in Table 1 seems to suggest a promising development of this library of derivatives against *V. cholerae* enzymes. In fact, although the moderate-low activity against *Vch*βCA and *Vch*γCA, a relevant inhibitory profile emerged for several compounds and, in some cases, a good selectivity on the bacterial α isoform with respect to the human ones is gained. Indeed, selectivity index (SI) values on *h*CA I reported in Table 1 show that the presence of a 4-chlorophenyl ring and the *n*-butyl tail are helpful to address the activity on *Vch*αCA for all the investigated scaffolds, while the tolyl group helps to gain selectivity only in the *meta*-series also towards the human isoform II. Considering the aromatic groups, the *h*CA I/*Vch*αCA selectivity is also reached by derivatives endowed with a phenyl ring on scaffolds **b** and **c** and the pyridinyl and benzyl functions in the *para*-series of scaffolds **a** and **c**. However, several compounds display a good *h*CA II/*Vch*αCA selectivity, even if with a different trend. Overall, the amino compound **10a**, the amido **4b**, and the ureidic **9c** and **12c** emerged as the most selective derivatives, with higher human/bacterial SI than **AAZ**.

We could argue that the increase in rigidity from the most flexible amino tail (scaffold **a**) to the amido (**b**) and cyclic urea (**c**) functions corresponds to an enhancement of the inhibitory activity of this class of compounds, maybe due to a different binding mode. However, clear evidence of the optimal sulfonamide moiety (*meta* or *para*) position on the structural nucleus could not be found from the *in vitro* assay results. In general, it is not trivial to draw accurate SARs with a large amount of experimental data, and a computational structure-based study of compounds into the binding pockets of the targeted enzymes could help the understanding of their inhibition and affinity.

### In silico studies

#### Flexibility properties calculation

The structural flexibility of studied ligands was assessed through the FAFDrugs4 webserver (FAFDrugs4, https://fafdrugs4.rpbs.univ-paris-diderot.fr/)^46^, considering the number of rigid and flexible bonds as established by Veber rules^47^. Examples of the calculated values are reported in Table 2.

**Table 2.** Flexibility properties of compounds **1a–c** and **9a–c**, selected as representative of the whole library of derivatives, calculated through FAFDrugs4 webtool.

CPD	Structure	Flexibility	Rotable bonds (#)	Rigid bonds (#)
**1a**	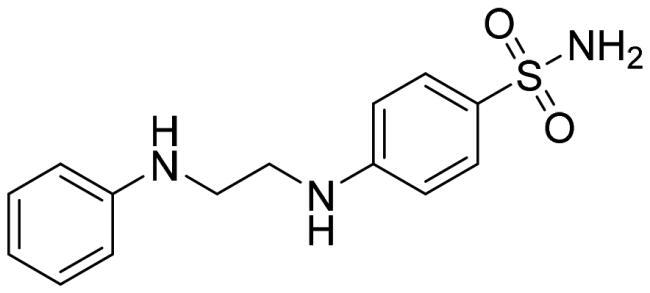	0.30	6	14
**1b**	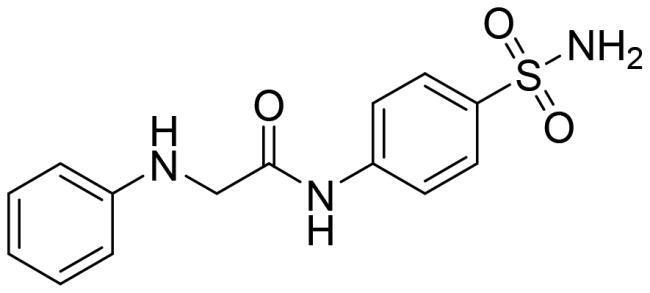	0.24	5	16
**1c**	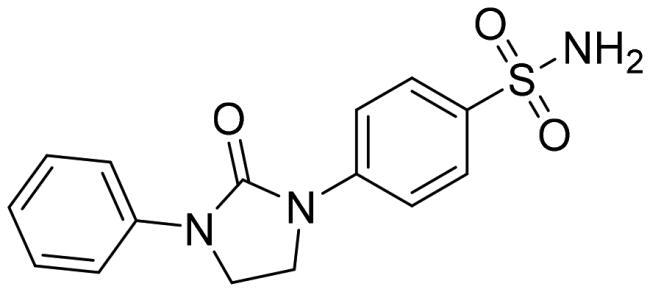	0.13	3	20
**9a**	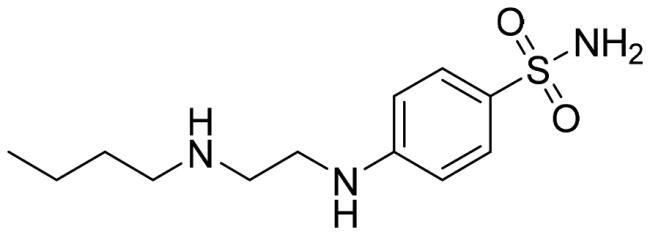	0.50	8	8
**9b**	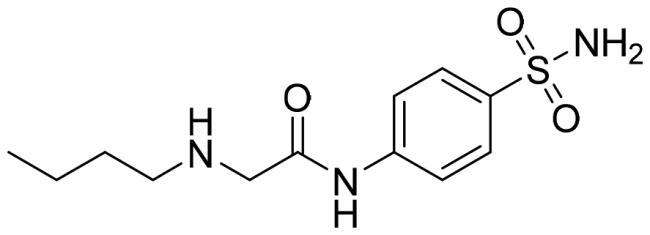	0.41	7	10
**9c**	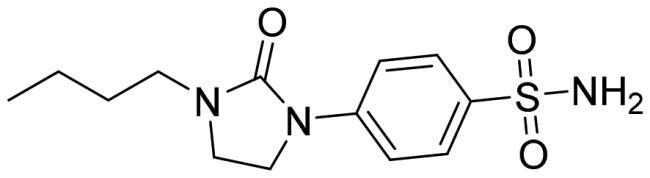	0.26	5	14

Observing data in Table 2, the same trend was noticed for both the phenyl substituted derivative **1** and the alkyl compound **9**, reported as representatives of the whole library of compounds. The flexibility value decreases within the series **a**–**b**–**c** from the amine (**a**) to the urea (**c**) in correspondence to the increase in the number of the rigid bond. However, very different values were obtained for **1** and **9**, since the aliphatic tail belonging to the latter is very flexible with respect to the aromatic ring of **1**. In fact, as expected, the alkyl derivatives **9a–c** hold a flexibility value higher than both **1b** and **1c**. In this study, we tried to better understand how this broad range of flexibility of the tails, also considering the different interactions they can establish with the enzyme residues, could affect the affinity and, thereby, the inhibitory activity of the compound.

#### Molecular docking and MD simulations on the VchαCA

To shed light on the binding mode of our benzenesulfonamide inhibitors, structure-based studies of all compounds in *Vch*CAs and *h*CAs were conducted.

Since the 3D coordinates of *Vch*αCA are not included in the PDB database, a homology modelling protocol has been applied. The *Vch*αCA model was based on the experimental coordinates of *Photobacterium profundus* (PDB: 5HPJ), showing the highest sequence identity (64.75%). During our study, the 3D structure of this protein predicted by AlphaFold2^48^ was made available in the AlphaFold Protein Structure Database (https://alphafold.ebi.ac.uk/) and was compared with the structure generated by homology modelling. The RMSD value for *Vch*αCA was 0.838 Å underpinning the validity of our model. To optimise residue positioning around the sulfonamide group, the AAZ geometry derived from the crystallographic complex with the αCA from *Helicobacter pylori* (PDB: 4YGF) was placed in the binding site after the proper alignment of the three zinc-coordinating histidine residues (His104, His106, and His123). To relax the protein with the known sulfonamide, the complex of the HM-*Vch*αCA and AAZ was minimised, and the obtained protein was used for the docking studies.

**Figure 3.**
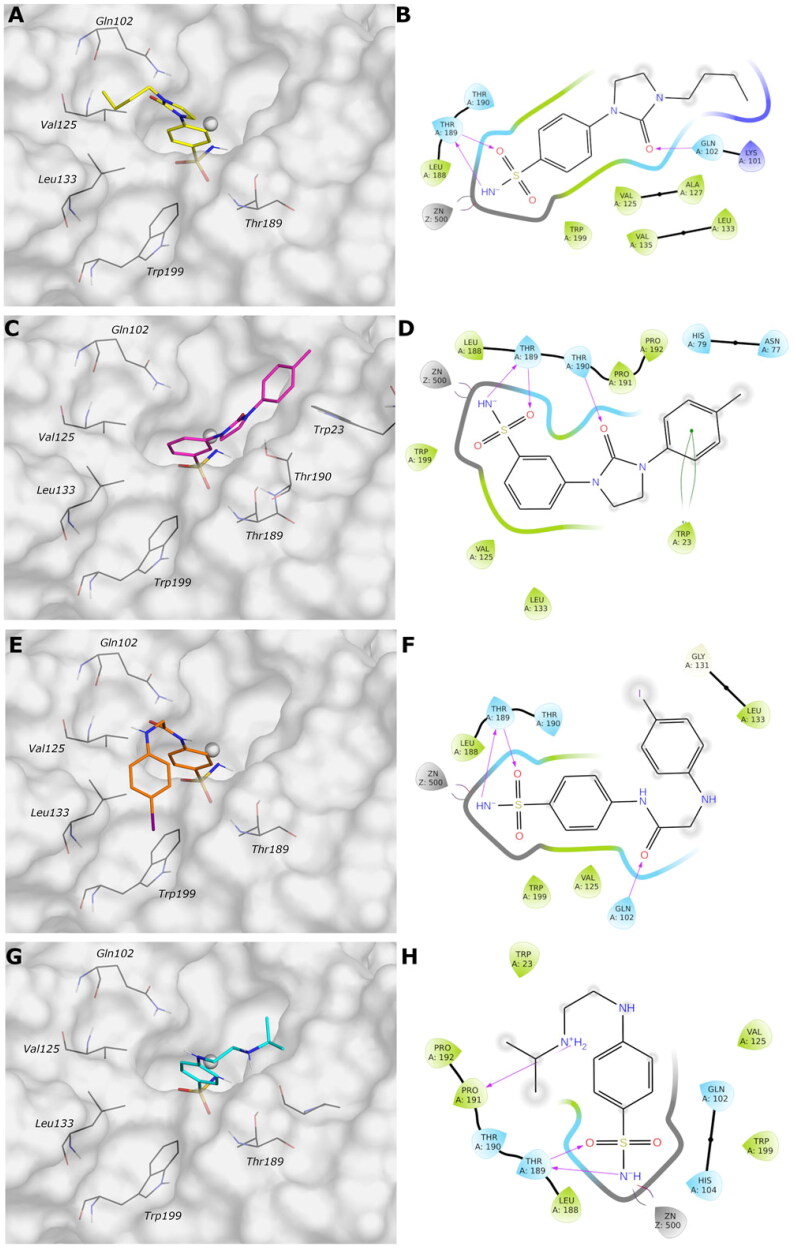
(A, C, E, and G) Predicted 3D binding mode and (B, D, F, and H) corresponding ligand interaction diagram of most selective ligands (A, B) **9c** yellow, (C, D) **12c** magenta, (E, F) **4b** orange, and (G, H) **10a** cyan, within *Vch*αCA (light grey). The compounds are represented as sticks, and the protein surface is visualised.

The docked poses reveal that all compounds assume a similar placement in the active site. The sulfonamide NH group, negatively charged, coordinates the zinc ion by a tetrahedral geometry and establishes an H-bond with the hydroxyl group of Thr189, while one of the sulphonyl oxygen H-bonds to the NH of the Thr189 backbone. We noticed a different binding mode depending on the position of the substituent: most *para*-substituted compounds point towards the *Vch*αCA coil (residues 128–132) (Figure 3(A,B,E–H)), while *meta*-substituted compounds move in the opposite direction (Figure 3(C,D)).

Considering the linker between the benzenesulfonamide portion and the R-group, more constrained compounds bearing the cyclic urea (**1**–**15c**) show a further H-bond between the cyclic carbonyl and, alternatively, Gln102 for *para*-substituted (**9c**, Figure 3(A,B)), or Thr190 backbone NH for *meta*-substituted (**12c**, Figure 3(C,D)). Compounds containing the amide linker show the same H-bond with Gln102 for *para*-sulfonamides, while the H-bond with Thr190 was conserved only for **4b** for *meta*-substituted (Figure 3(E,F)).

**Figure 4.**
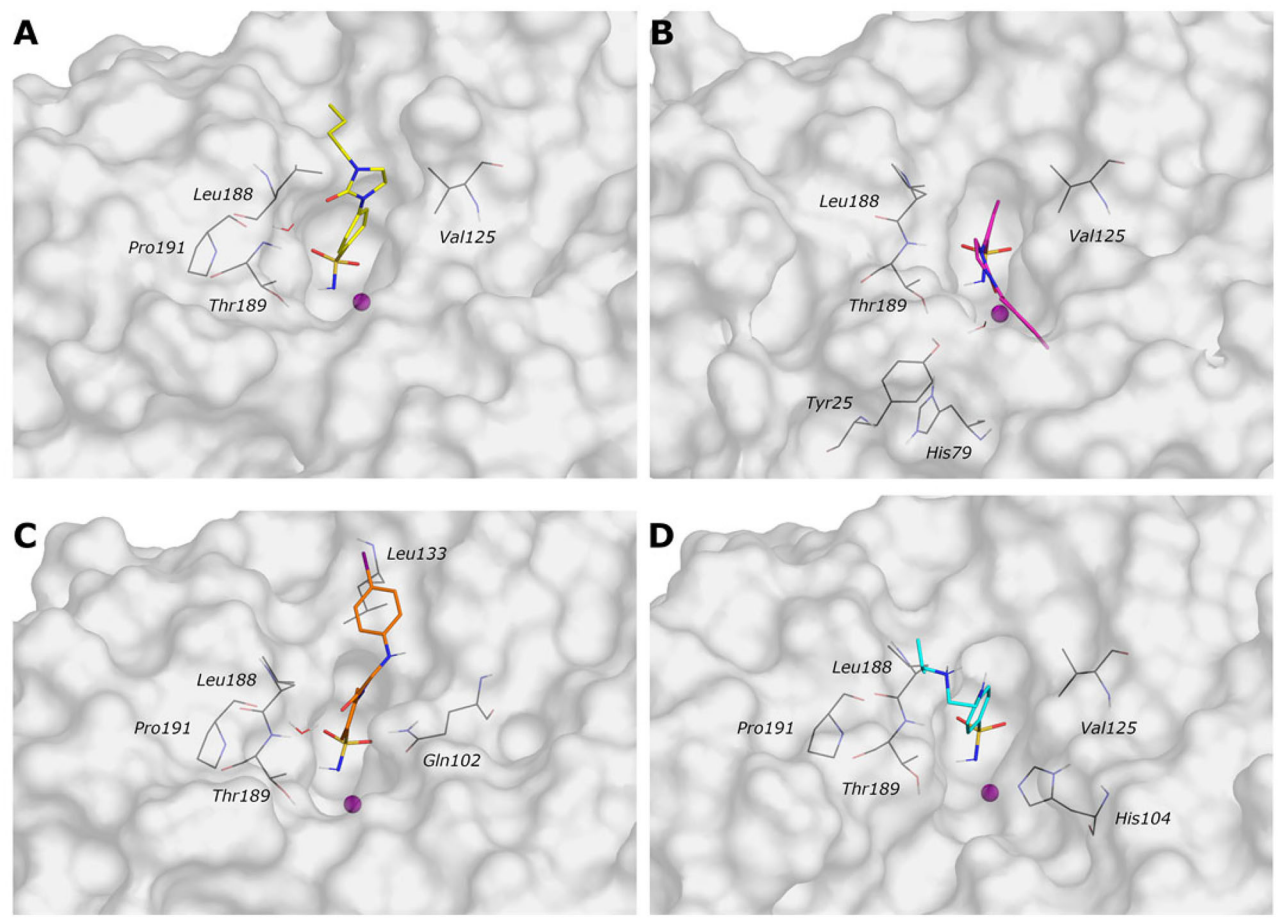
Most representative geometry retrieved after MD simulation for the four studied ligands. (A) **9c** yellow, (B) **12c** magenta, (C) **4b** orange, and (D) **10a** cyan in the *Vch*αCA (light grey). The compounds are represented in stick and the protein surface is visualised.

In the *para*-substituted series with the more flexible linker, the amine group, only compound **10a** (Figure 3(G,H)) makes an additional H-bond between the distal amine and the Pro190 carbonyl, preferring a different placement on the active site with respect to the other *para*-compounds. To obtain more insight into the binding process, 100 ns MD simulations on the *Vch*αCA complexes with most selective inhibitors **9c**, **12c**, **4b**, and **10a** were carried out (Figure 4(A–D)).

MD simulation analysis highlights the good stability of the studied complexes. The RMSD values calculated on the protein *Vch*αCA are relatively stable along the simulation and lower than 2.5 Å for all complexes. The same parameter calculated for ligands shows larger values, indicating a change in all ligands’ initial binding conformation maintained along the production phase. The distal substituent to the benzene ring, exposed to the solvent, is the less stable part of the molecules during the simulation and is in charge of raising the RMSD value of the ligands as underlined by the RMSF values (Figures S1–S2 in Supporting Information). On the contrary, the benzenesulfonamide portion of all analysed ligands is firmly bound to the zinc ion and to Thr189 via a hydrogen bond. The most constrained compounds, **9c** (*K*_I_ = 4.7 nM) and **12c** (*K*_I_ = 11 nM), do not establish a π–π stacking contact with the zinc-coordinated His104 as for **4b** and **10a**, probably due to the steric hindrance of the 1,3-imidazolidin-2-one, but the carbonyl urea finds a water bridge interaction with Pro191 (Thr190) or Tyr25.

**Figure 5.**
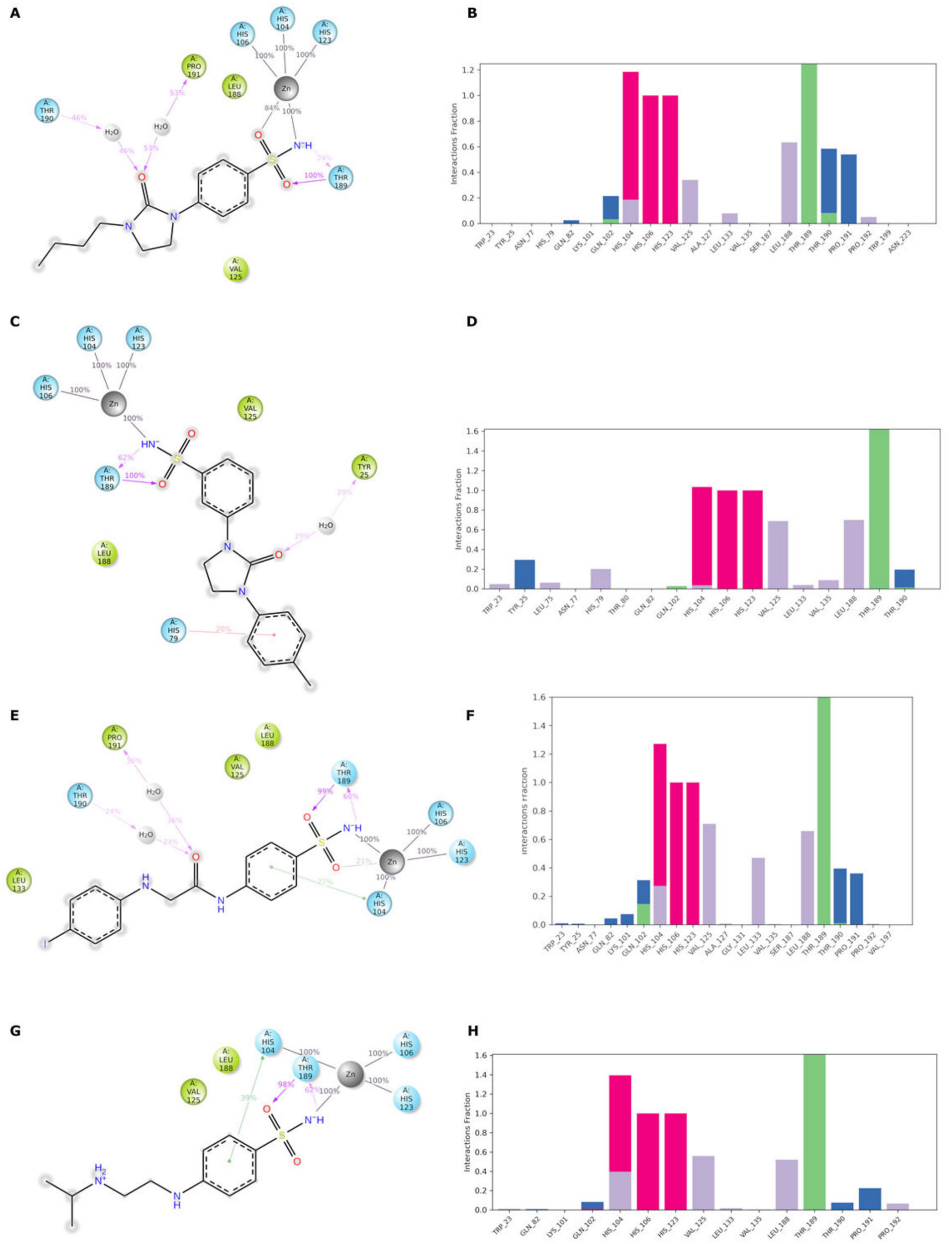
(A, C, E, and G) The 2D representation of most conserved ligand–protein interactions with (B, D, F, and H) the indication of the persistence (%) along the simulation and depiction of frequency and type of ligand-protein interaction along with the MD simulation. (A, B) **9c**, (C, D) **12c**, (E, F) **4b**, and (G, H) **10a**.

For ligand **9c** (Figure 4(A)), the protein average RMSD is close to 1.4 Å, whereas the ligand RMSD is 2.8 Å. During the simulation, the H-bond of the carbonyl urea with Gln102 is maintained at a very low percentage and is replaced by a water-bridged interaction with persistence of 22% (Figure 5(A)). The 1,3-imidazolidin-2-one turns in the opposite direction of Gln102 to find a water bridge interaction between the carbonyl oxygen and Pro191 (53%) or Thr190 (46%), confirming its conserved involvement in HB interactions even with different residues. Few hydrophobic interactions with residues of Val125, Leu133, and Leu188 are present in line with the docking study, and the *n*-butyl portion is exposed to the solvent (Figure 5(A,B)).

The analysis of the MD simulation of ligand **12c** (Figure 4(B)) reveals an average protein RMSD of 1.6 Å, whereas the average ligand RMSD is 2.6 Å. Similar to the previous inhibitor, the H-bond between the carbonyl group and Thr190 is maintained only for 20% of the simulation, while the 1,3-imidazolidin-2-one slightly turns to find a water bridge with Tyr25 (29%). The π-π stacking contact between the distal phenyl and Trp23 is similarly maintained for a very short time during the simulation, and it is replaced by a π-π stacking contact with His79 (20%, Figure 5(C,D)), following the rotation of the linker.

For ligand **4b** (Figure 4(C)), the average protein RMSD is 1.5 Å, whereas the ligand RMSD is 2.5 Å, with a significant fluctuation during the simulation caused mainly by the rotation of the 4-iodophenyl ring that flips between two different positions, as highlighted by the RMSD profile. As for the previous compounds, the benzenesulfonamide function coordinates the zinc ion, but there is also evidence of a π-π stacking between the benzene ring and His104 (27%). The carbonyl amide forms a water bridge interaction with Pro191 (36%) or Thr190 (24%), while the interaction with Gln102 is maintained for less than 20% of the production phase (Figure 5(E,F)), as for compound **9c**. The distal portion of the molecule is exposed to the solvent.

For the less restricted compound **10a** (Figure 4(D)), the protein average RMSD is 1.7 Å, whereas the ligand RMSD is 2.1 Å, with the benzene sulfonamide substituent moving quite enough during the simulation. Other than the expected interactions of the sulfonamide with the zinc ion and Thr189, a π–π stacking contact between the benzene ring and His104 is present, also seen in the compound with the amide linker (**4b**). The interaction between the distal amine and Pro191 found in the docking study is maintained only for 20% of the simulation. The few hydrophobic interactions involve the residues Val125 and Leu188, whereas the linker and the isopropyl remain exposed to the solvent (Figure 5(G,H)).

#### Docking on the hCA I and hCA II

One of the current project’s key aspects is the identification of compounds that selectively inhibit *Vch*CAs, sparing *h*CAs. To find out the critical features for the selectivity over, in particular, the *Vch*αCA, a docking protocol has been carried out on the human CA isoforms I and II (PDB ID: 6IOJ and 3K34, resolution 1.35 and 0.90 Å, respectively).

The analysis of the docking poses reveals that all compounds successfully adopt the correct geometry of the sulfonamide function that coordinates the zinc ion and a π–π stacking contact of the phenyl ring with His94 (*h*CA I and *h*CA II) for most compounds. As for *Vch*αCA, hydrophobic interactions are not prevalent, and the linker and the tail of most compounds are exposed to the solvent.

**Figure 6.**
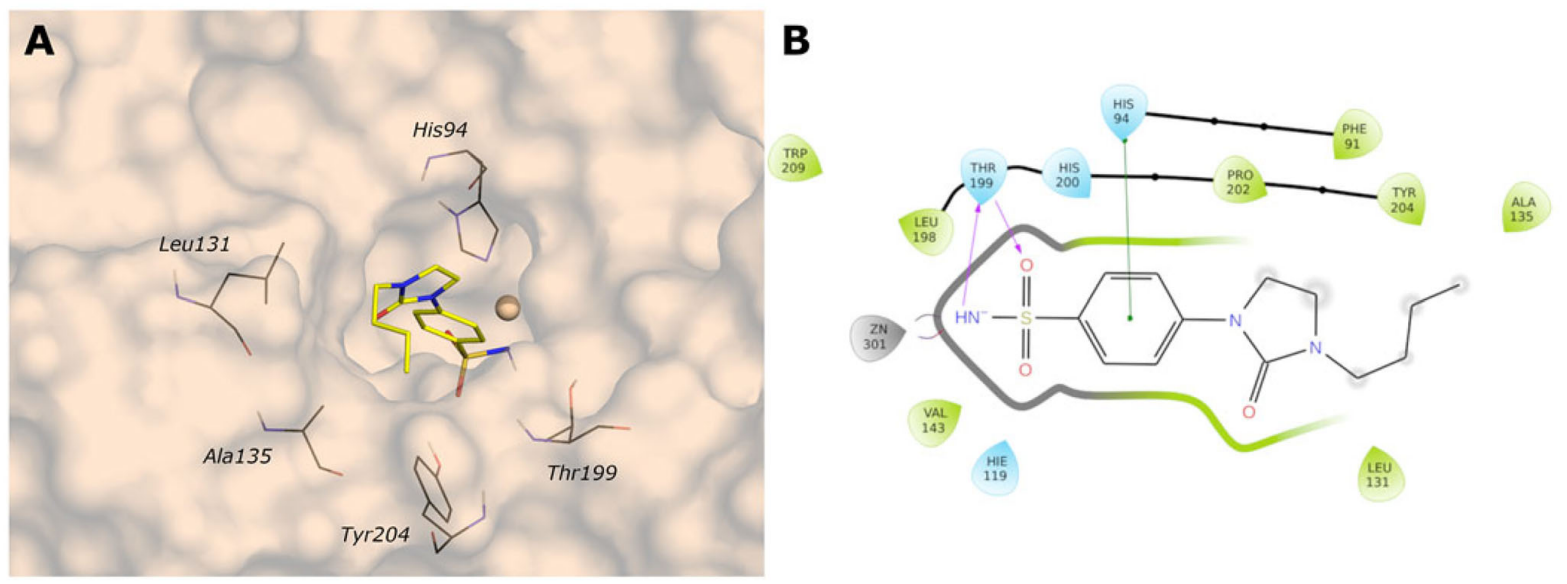
(A) Predicted 3D binding mode and (B) corresponding ligand interaction diagram of the most selective ligands **9c** (yellow) in the *h*CA I (light salon). The compound is represented as the stick and the protein surface is visualised.

**Figure 7.**
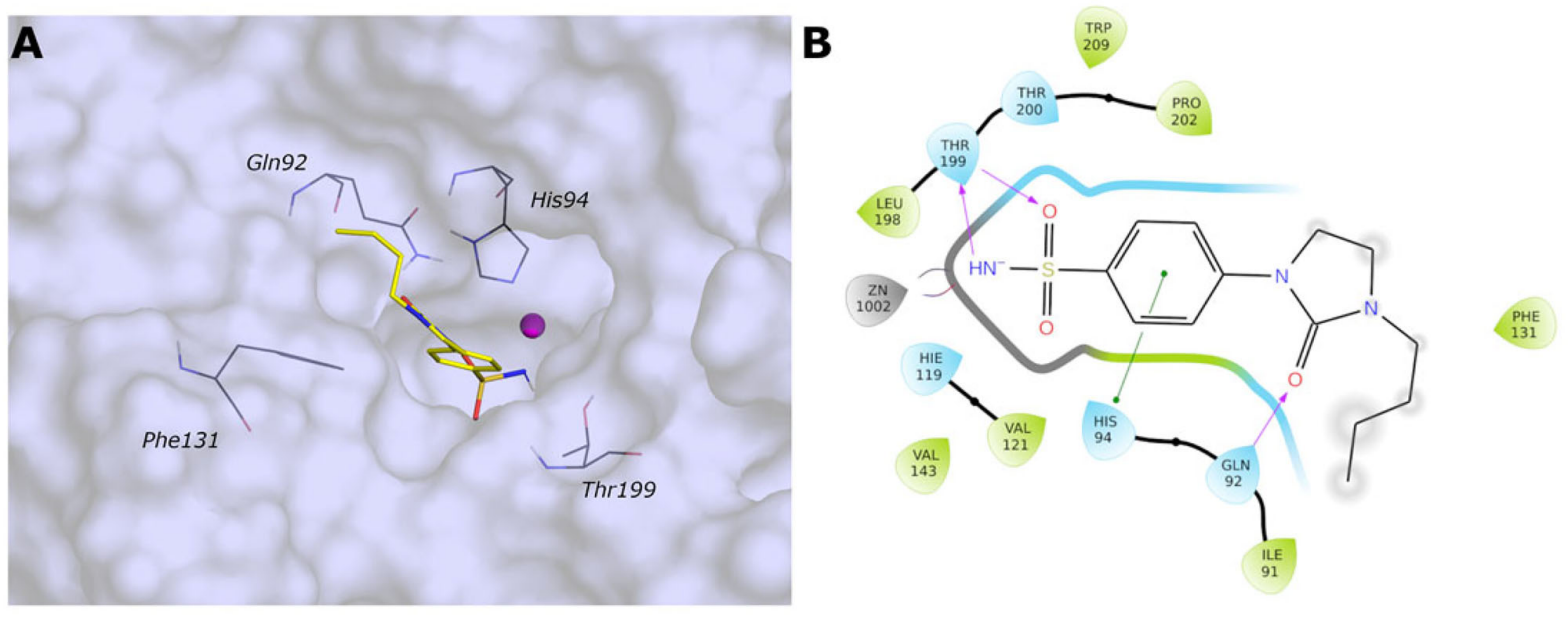
(A) Predicted 3D binding mode and (B) corresponding ligand interaction diagram of the most selective ligand **9c** (yellow) in the *h*CA II (light cyan). The compounds are represented as a stick and the protein surface is visualised.

The active sites of hCA I and hCA II are superimposable to the active site of *Vch*αCA, except for the α-helix comprising residues 124–140 for *h*CA I and *h*CA II, which is much shorter in *Vch*αCA and forms a coil (residues 128–132)^49^. This determines a partial restriction at the edge of the active site of *h*CAs, causing a different placement of the inhibitor tail (Figures 6 and 7, Figures S3 and S4).

#### Docking on the VchβCA

Even if the activity of compounds results in the micromolar order on the *Vch*βCA, we investigated the binding mode of the studied compounds in the narrower active site of this isoform. Starting from the coordinates of the “closed” form of *Vch*βCA (PDB: 5CXK), the “open” form was generated by trimming the fundamental residue Asp^A^44 during the Induced Fit protocol to obtain the conformational changes in the target sidechain that can resemble the “open” conformation of the *Vch*βCA. The obtained conformation of this residue was verified by overlapping the obtained protein with the crystal structure of the “open” β-CA of *Aspergillus fumigatus* (PDB: 7COJ). The coordinates of the cocrystallized **AAZ** in complex with β-CA of *Coccomyxa* (PDB: 3UCJ) were used to minimise the obtained “open” form of *Vch*βCA and optimise the residue positioning around the sulfonamide function. The minimised protein was then used to dock all ligands into the active site.

The docked poses of all compounds confirm that the sulfonamide group is coordinated and correctly oriented to the zinc ion, and the benzene ring interacts with Tyr^B^83 by π–π stacking, reproducing the typical orientation of benzenesulfonamide inhibitors. Other fundamental contacts are with the hydrophobic pocket at chains A and B interface, involving Gly^A^102, Gly^A^103, Ala^A^106, Pro^A^113, and Leu^A^113.

**Figure 8.**
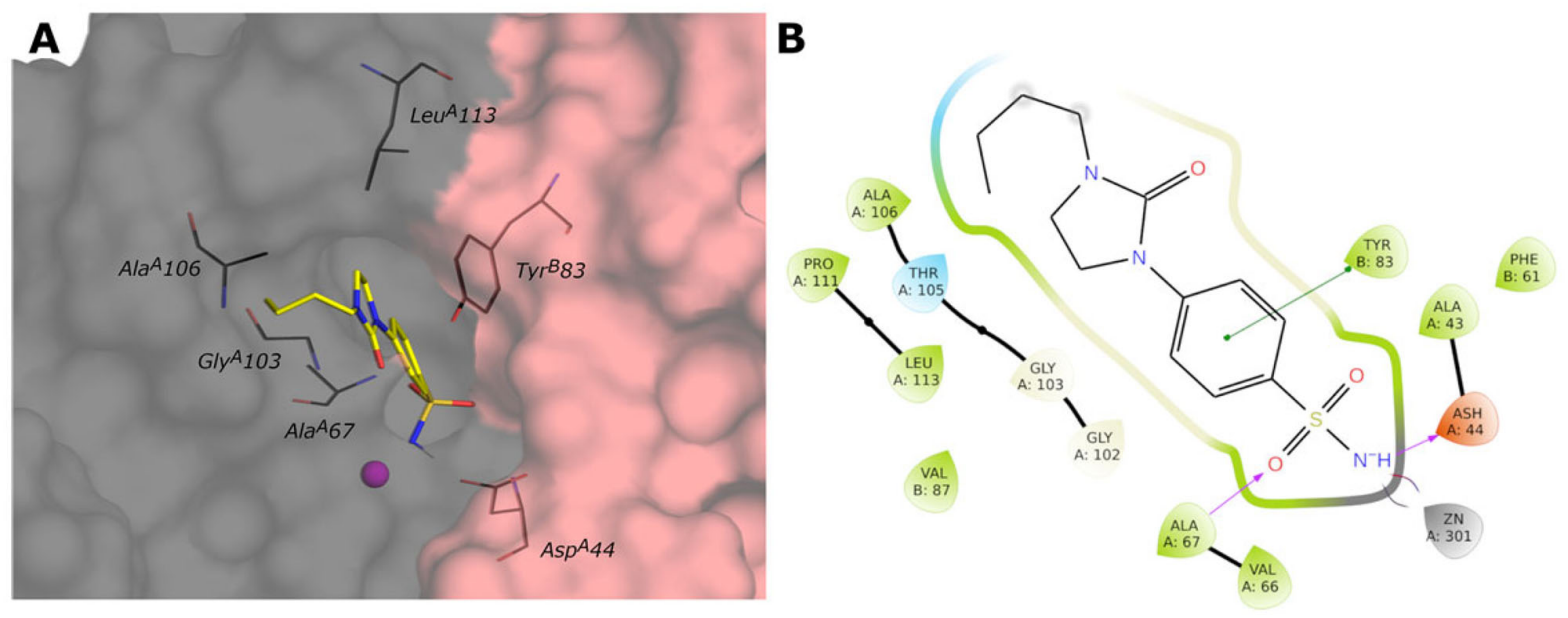
(A) Predicted 3D binding mode and (B) corresponding ligand interaction diagram of the most selective ligand **9c** (yellow) within *Vch*βCA (grey and pink). The compound is represented as a stick and the protein surface is visualised.

The docking results do not demonstrate different poses depending on the type of linker (amine, amide, or cyclic urea) or the *para* or *meta* position of the R-group. Even though the correct placement of the benzenesulfonamide group in the tight cleft of the *Vch*βCA, none of the compounds can establish more favourable interactions further than hydrophobic. The predicted binding mode and the ligand interactions of **9c** within the *Vch*βCA active site are represented in Figure 8.

#### Docking on the VchγCA

The 3D structure of *Vch*γCA was generated by homology modelling using the 3D crystal structure of γ-CA from *Escherichia coli* (PDB: 3TIO, identity 64.16%) as the template. The active site, located at the interface of two chains of the trimer of the *Vch*γCA, can exist in the “open” or “closed” conformation as already observed for the β isoform. The open form between chains D and F of 3TIO was chosen to model corresponding chains of the homologous *Vch*γCA. The 3D structure produced by homology modelling was compared with that predicted by AlphaFold2^48^ and is available in the AlphaFold Protein Structure Database (https://alphafold.ebi.ac.uk/). Also in this case, the validity of our model was supported by the low RMSD value (0.772 Å) obtained by superimposing the two *Vch*γCA structures. As no benzenesulfonamide X-ray ligands are known to bind γ-CAs, we used the previously studied 4-(hydroxymethyl)benzenesulfonamide^31^ as a reference to validate our model and optimise residues surrounding the sulfonamide portion. This compound was docked into *Vch*γCA, and the best-docked pose reveals the correct coordination geometry with the zinc ion, two H-bonds between the sulfonamide group and Gln^F^61, the H-bond between the hydroxyl and Asp^D^114, and hydrophobic interaction with Leu^D^113, Met^F^108, Leu^F^105, Tyr^F^168, similar to those previously obtained^31^. The complex of the best-docked pose of 4-(hydroxymethyl)benzenesulfonamide and the HM-*Vch*γCA was minimised to optimise residue positioning around the ligand. The obtained protein was then used to dock all compounds. The binding mode of all sulfonamide inhibitors in the active site replicates the interaction with zinc ion and Gln^F^61. For compounds containing the amine linker (**1–15a**), the residue Asp^D^114 is involved in an H-bond with the distal or proximal amine of ligands of the series *meta-* and *para*-substituted.

**Figure 9.**
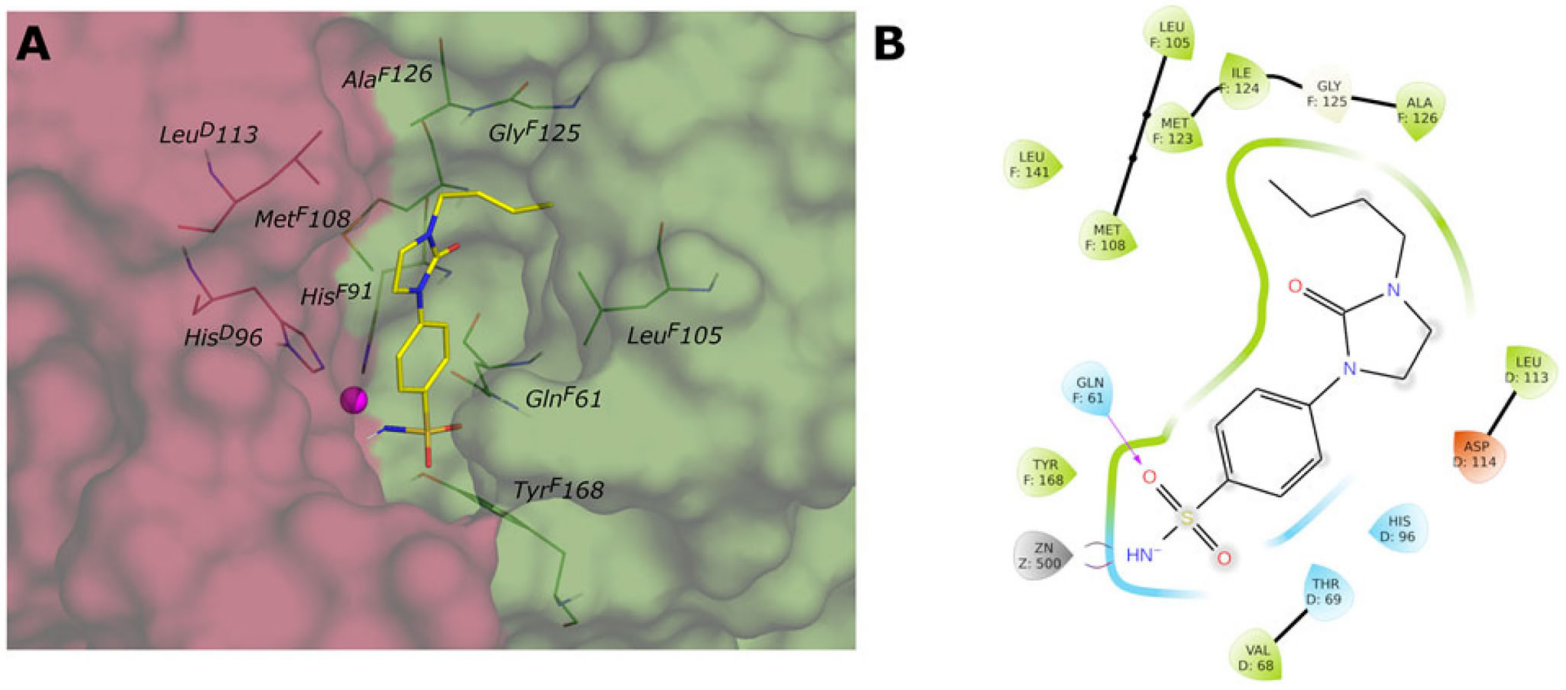
(A) Predicted 3D binding mode and (B) corresponding ligand interaction diagram of the most selective ligand **9c** (yellow) within *Vch*βCA (grey and pink). The compound is represented as a stick and the protein surface is visualised.

The *para*-substituted inhibitors with the amide linker (**1–15b**) deploy in the cylindrical active site interacting with the Met^F^108 NH by the carbonyl group of the amide linker or with the carboxyl group of Asp^D^114 by the NH of the amide. Only compound **15b** of the *meta*-substituted inhibitors interact with Asp^D^114 with the amide and amine groups. The inhibitors characterised by the cyclic urea, in addition to the interactions of the sulfonamide group, engage only hydrophobic contacts with Leu^F^105, Gly^F^107, Met^F^108, Leu^F^141, Met^F^143, Leu^D^113, Pro^D^132. Figure 9 shows the predicted binding mode and the ligand interactions of ligand **9c** within the *Vch*γCA active site.

**Figure 10.**
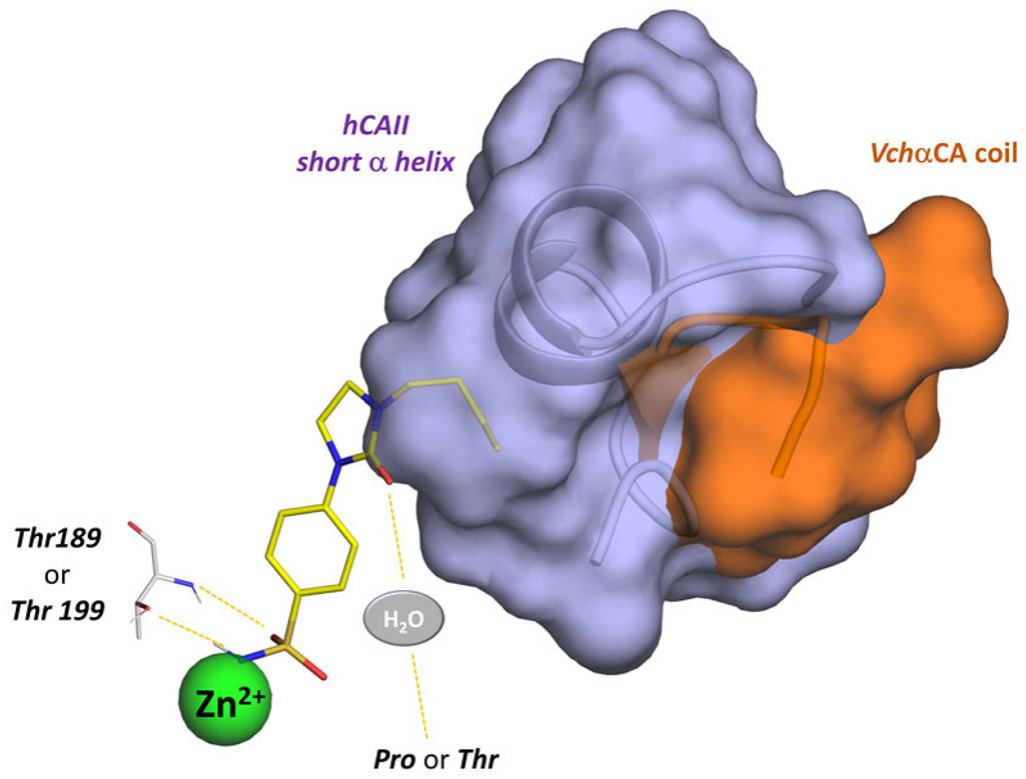
Schematic representation of the ligand contacts producing the selectivity towards *Vchα*CA with respect to *h*CAs. The compound (**9c**) is represented as sticks and the *Vchα*CA coil and the short alpha helix *h*CAII (similar to *h*CAI) are visualised as cartoon and surface.

In summary, the computational analysis suggests that the structural determinants contributing to ligand selectivity towards *Vchα*CA with respect to *h*CAs lie in the tail portion of arylsulfonamide ligands. Arylsulfonamides are well-known binders of αCAs, interacting with the zinc ion via the negatively charged NH^−^ group and establishing well-conserved H-bonds with a threonine residue. On the other hand, the rigidity of the linker increases the binding affinity towards all αCAs, both the human and bacterial isoenzymes. A more promising activity profile is accompanied by an H-bond acceptor in the rigid linker that directs the tail portion towards the *Vchα*CA coil (residues 128–134). The corresponding region in the human CAs is occupied by a short α-helix comprising residues 121–140 and constituting the most diverse region between human and bacterial αCAs (Figure 10).

#### Physicochemical and pharmacokinetic properties calculation

Parameters affecting the drug-likeness and bioavailability of the studied sulfonamides were predicted by using QikProp calculations^50^ (Table S1 in Supporting Information).

Considering these compounds should act locally in the intestinal tract, we focussed on properties favouring their low absorption. It is known from the literature that compounds working locally in the gut^51^ are usually polar, with a high molecular weight (MW) and polar surface area (PSA). In this case, focussing on the most selective inhibitors, it is possible to highlight their compliance with Lipinski’s Rule of five, their limited permeability of the gut–blood barrier, and the blood-brain barrier (BBB). Some concerns are due to the possible binding to HERG. Table 3 shows the essential properties for the most selective compounds.

**Table 3.** Physicochemical and pharmacokinetic properties of the studied ligands.

cpd	MW	PSA	Rule of Five	% *h* Oral Abs	QPP Caco	QP logBB	QP logHERG
**9c**	297.371	95.123	0	76.996	209.196	−1.454	−4.882
**10a**	257.35	89.372	0	60.538	67.277	−1.064	−5.688
**12c**	331.389	93.581	0	80.227	228.807	−1.242	−5.54
**14b**	384.247	111.98	0	72.068	134.213	−1.639	−6.072
**AAZ**	222.236	133.243	0	44.375	35.654	−1.802	−3.791

MW: molecular weight; PSA: Van der Waals surface area of polar nitrogen and oxygen atoms and carbonyl carbon atoms (7–200); Rule Of Five: Number of violations of Lipinski rule of five; *h* Oral Abs: human oral absorption (1, 2, or 3 for low, medium, or high); % *h* OralAbs: Predicted human oral absorption on a 0–100% scale; QPPCaco: Predicted apparent Caco-2 cell permeability in nm/s. Caco-2 cells are a model for the gut–blood barrier (500 great); QPlogBB: Predicted brain/blood partition coefficient (−3.0 to 1.2); QPlogHERG: Predicted IC_50_ value for the blockage of HERG K^+^ channels (concern below −5).

### Direct-acting antibacterial activity

Most of the compounds (**1a**, **2c**, **3a–c**, **4–5c**, **6b–c**, **7c**, **8–9a–c**, **10c**, **11a–c**, **12c**, **13a–c**, **14a**, **15a**) and **AAZ** were tested on three different clinical isolates of *V. cholerae*, namely SI-Vc22, SI-Vc71, and SI-Vc912, to assess their direct antibacterial activity. Using an agar diffusion method (ampicillin, chloramphenicol, and ciprofloxacin were used as reference antibiotics), only twelve (amines **8–9a**, **11a**, and **13a**, amides **3b**, **9b,** and **11b**, ureas **4–7c** and **9c**) out of 28 tested compounds showed a moderate growth inhibition against at least one of the clinical isolates. Five compounds (amines **9a**, **11a,** and ureas **4c**, **6c**, **9c**) were found to be moderately active on all the isolates (inhibition zone diameter in the range of 3–5 mm; data not shown).

However, the determination of the minimum inhibitory concentrations (MICs) (broth microdilution method) confirmed the very low direct-acting activity of these compounds, as MIC values were ≥64 µg/mL. These data are not entirely unexpected considering that the inhibition of *Vch*CAs would not result to be to be detrimental for the bacterium *in vitro*, but would be important *in vivo*, especially for the onset of virulence, whose study would require far more complicated biological investigations. However, and since we can now confirm the lack of a direct antibacterial activity for all the derivatives, it would be extremely interesting to assess whether these compounds could inhibit the production of the active exotoxin (e.g. with gene reporting, biochemical or cellular assays) to verify this hypothesis^52^.

## Conclusions

Herein, we reported the design strategy of CA inhibitors bearing conformationally restricted alkyl/aryl amines and amides into imidazolidinones. These *para-* or *meta*-benzenesulfonamides are *ad hoc* characterised by differential rotational features, leading to highly potent (nanomolar) and selective compounds acting preferentially against *Vch*αCA with respect to β- and γ-isoforms.

Stopped flow-based enzymatic assays showed that all the compounds, with properly *inter*-series differences, are able to strongly inhibit *Vch*αCA, with the following progressive inhibitory potency: *meta*-benzenesulfonamides < *para*-benzenesulfonamides and amine < amide < cyclic urea.

Although the above-mentioned trend was not completely respected and it resulted to be quite different for some substituents, we could notice that the more the linker is rigid (urea) the more the inhibition is strong. Thus, by performing these preliminary SAR considerations, we could assume that the activity profile of the derivatives library is strictly linked to the different rigidity of the compounds tail. After a preliminary calculation of the flexibility properties of representative compounds, we find out how they affect the affinity of the whole set of compounds for the different enzyme catalytic sites and, thereby, their inhibitory activities. In particular, we investigated their binding poses in both the human and bacterial enzymes through an in-depth structure-based computational study. Moreover, MD simulations of the most selective inhibitors of *Vch*αCA revealed the high stability of the benzenesulfonamide core in the interaction with the zinc ion, assuming the usual coordination geometry. The structural determinants able to guarantee a proper interaction with the active site were found to be (i) the presence of the urea carbonyl group in the linker (**9c**), able to establish a water-mediated hydrogen bond with Pro191 or Thr190, and (ii) the constraint of the linker in cyclic urea, producing the highest selectivity over the *h*CA I and II.

*In silico,* ADME was also investigated to assess the physicochemical properties of the benzenesulfonamide derivatives and the compounds were found to be in accordance with Lipinski’s rule, even if they seem to suffer from a low oral bioavailability. Keeping into consideration the localisation of our target, this property can be considered fruitful to avoid systemic distribution and off-target interaction.

Although not entirely unexpected, antibacterial susceptibility assays on three different epidemiologically unrelated *V. cholerae* strains confirmed the lack of direct-acting antibacterial activity of these compounds. Further characterisation of these compounds would be required to evaluate their potential inhibition of the production of virulence determinants, of primary importance in the host environment, although this study is beyond the scope of the present work.

In summary, a combined analysis of inhibitory enzymatic activity and *in silico* simulations of a large library of benzenesulfonamide derivatives revealed and rationalised specific SARs regarding the rigidity/flexibility properties of the compounds tail. Thus, thanks to our protocol, we screened 45 benzenesulfonamides decorated with three different moieties (amine, amide, and cyclic urea) and a number of decorated tails against the three CA expressed by *V. cholerae* pathogen though the versatile and high-throughput stopped-flow technique. The obtained *K_I_* values highlighted interesting activity profile for several derivatives and notable isoform selectivity was found for at least one compound for series, such as the 4-benzenesulfonamides with amino (**10a**), amido (**4b**), and the cyclic urea (**9c**) moieties and only one 3-benzenesulfonamide derivative (**12c**).

Further efforts will be focussed on the assessment of the antibacterial behaviour of these compounds in an infection model to evaluate their activity towards the virulence and pathogenicity of the bacterium.

## Material and methods

### Preparation of the derivative libraries

**Scheme 1.**
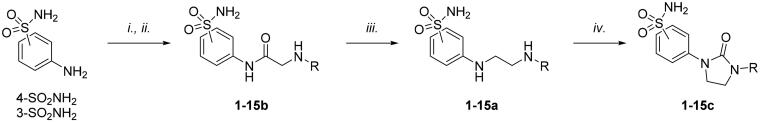
Synthesis of compounds **1-15a–c**. Reagents and conditions: (i) chloroacetyl chloride, dry acetone, N_2_, 0 °C, 0.5 h; (ii) appropriately substituted aniline, KI, sealed tube, dry THF, N_2_, 110 °C, 24 h; or 2-amino-6-methylpyridine, dry TEA, abs EtOH, N_2_, ref., 24 h; or benzylamine, dry TEA, dry ACN, N_2_, 24 h; or amine, KI, dry THF, N_2_, 24 h; (iii) 1 M BH3·THF, dry THF, N_2_, r.t., 24 h; or LiAlH_4_, dry THF, N_2_, 0–70 °C, 24 h; (iv). triphosgene, dry TEA, dry THF, N_2_, r.t., 2 h.

The compounds were prepared as reported according to the general synthetic approach reported in Scheme 1^40^.

### In vitro carbonic anhydrase inhibition assay

The CA-catalyzed CO_2_ hydration activity was performed on an Applied Photophysics stopped-flow instrument using Phenol Red, at a concentration of 0.2 mM, as a pH indicator working at the maximum absorbance of 557 nm^53^^,^^54^ with 20 mM HEPES (pH 7.40 for α- and 8.40 for β-CAs and γ-CAs) as the buffer, 20 mM Na_2_SO_4_ to maintain constant ionic strength, and following the initial rates of the CA-catalyzed CO_2_ hydration reaction for a period of 10 − 100 s and The CO_2_ concentrations ranged from 1.7 to 17 mM for the determination of the kinetic parameters and inhibition constants. Enzyme concentrations ranged between 5 and 12 nM. For each inhibitor, at least six traces of the initial 5 − 10% of the reaction have been used to determine the initial velocity. The uncatalyzed reaction rates were determined in the same manner and subtracted from the total observed rates. Stock solutions of inhibitor (0.1 mM) were prepared in distilled-deionized water, and dilutions up to 0.01 nM were prepared. Solutions containing inhibitor and enzyme were preincubated for 15 min at room temperature prior to performing the assay to allow the formation of the E − I complex. The inhibition constants were obtained by nonlinear least-squares methods using PRISM 3^29^ and the Cheng-Prusoff equation as reported earlier and represent the mean from at least three different determinations. All CA isoforms are recombinant and obtained *in-house*, as reported earlier^29^.

### In silico studies

#### Calculation of flexibility properties of the derivatives

Flexibility and numbers of rotable/rigid bonds were calculated through FAFDrugs4 (Free ADME-Tox Filtering Tool)^46^.

#### 3D protein structure retrieval

Molecular modelling studies were performed on Schrödinger Life-Sciences Suite 2021–4^50^. All ligands were drawn as 2D structures from Maestro and prepared by using LigPrep to generate the 3D geometry and find all possible tautomers and protonation states at pH 7.0 ± 0.4 with Epik^55^^,^^56^. The three-dimensional X-ray structures of *h*CA I and *h*CA II were retrieved from the Protein Data Bank (PDB ID: 6IOJ and 3K34, resolution 1.35 and 0.90 Å, respectively)^57^^,^^58^. The Protein Preparation workflow was used to correct, optimise and minimise the crystal structures. The crystal structure of *Vch*βCA was retrieved from the Protein Data Bank (PDB ID: 5CXK, resolution 1.90 Å)^59^. The “open” catalytic site of *Vch*βCA was realised starting from the crystallographic “closed” form of the crystal structure. The Induced-fit protocol^60–62^ was used by employing Glide^63–65^ and Prime^66^^,^^67^ software with the OLPS4 force field, and the fundamental residue Asp^A^44 was trimmed to obtain the conformational changes in the target sidechain that can resemble the “open” conformation of the *Vch*βCA. The resulting conformation of the Asp^A^44 side chain was checked by overlapping it to the “open” *Vch*βCA of the fungal pathogen *Aspergillus fumigatus* (PDB: 7COJ)^68^.

#### Homology modelling

The 3D structures of *Vch*αCA and *Vch*γCA were obtained by homology modelling. The primary sequences of *Vch*αCA (Uniprot ID: A0A0H6VI20, 249 aa) and *VCh*γCA (Uniprot ID: A0A0H6TVJ0, 184 aa) were retrieved from the UniProt KnowledgeBase (UniProtKB) database^69^. The protein sequence was used as a query sequence for homology modelling using Prime. This tool used BLAST^70^ to identify suitable templates from Protein Data Bank (PDB) using a single template protocol. The homology models of *Vch*αCA and *Vch*γCA were obtained on the structure of αCA from *Photobacterium profundus* (PDB: 5HPJ, identity 64.75%) and the structure of γ-CA from *Escherichia coli* γCA (PDB: 3TIO, identity 64.16%) as the template, respectively^71^^,^^72^. For *Vch*γCA, the chains D-F were used as the template as they present the catalytic site in the “open” form. The crystallographic **AAZ** ligand coordinates taken from the complex with *Helicobacter pylori* αCA (PDB: 4YGF) and the 4-(hydroxymethyl)benzenesulfonamide^31^ were added as ligands to the homology models of α and γ isoforms, respectively. The protein-ligand complexes were fully minimised by using MacroModel applying the OPLS4 force field, 5000 steps of PRCG minimisation algorithm with a convergence criterion of 0.05 KJ/mol Å, to optimise the residue positioning around the sulfonamide function.

#### Docking calculations

Molecular docking analyses were performed using the Glide software. The Glide Grids were generated by positioning the enclosing boxes on the centre of mass of the respective sulfonamide ligands. The SP docking protocol was used by setting 5000 poses per ligand for the initial phase and 400 poses per ligand for energy minimisation with the OPLS4 forcefield. Rotatable groups were defined for each protein: Thr189 for *Vch*αCA, Asp^A^44 for *Vch*βCA, Thr^D^69 for *Vch*γCA, and Thr199 for *h*CAs. Additionally, a core constraint on the sulfonamide positioning was applied in the docking protocol into the *Vch*αCA and *Vch*βCA.

#### Molecular Dynamics

Molecular Dynamics simulation was carried out using Desmond^50^^,^^73^. The complexes of *Vch*αCA with the docked poses of compounds **9c**, **12c**, **4b**, and **10a** were embedded in an orthorhombic box of TIP4P water molecules resulting in systems of 28 787, 28 824, 28 748, 28 840 atoms, respectively. In order to balance the system charge, sodium, and chlorine ions were added. The systems were relaxed by applying the default relaxation protocol before the production phase. The simulation duration was set to 100 ns registering frames every 100 ps. The OPLS4 force field, a normal pressure-temperature (NPT) ensemble with a Nose–Hoover thermostat set to 300 K and a Martyna–Tobias–Klein barostat set to 1.01325 bar pressure were used. Electrostatic interactions were examined by applying the smooth particle mesh Ewald method. Figures were generated using Maestro and PyMoL^74^.

#### QikProp calculations

Physico-chemical and pharmacokinetic parameters were calculated using QikProp^50^ and applying the default parameters.

### Antibacterial susceptibility testing

Epidemiologically unrelated clinical isolates of *V. cholerae* (SI-Vc22, SI-Vc71, and SI-Vc-912) were obtained from the collection of the Department of Medical Biotechnologies (University of Siena, Italy). Compounds were resuspended in DMSO at a final concentration of 50 mg/mL. For insoluble compounds, DMSO was further added to lower the concentration until complete solubility of the compound (final concentrations, 25, 12.5, or 6.25 mg/mL). The direct antibacterial activity was evaluated using an agar diffusion-based method^75^. Briefly, Mueller-Hinton agar plates were inoculated with a bacterial suspension containing ≈1.5 × 10^8^ CFU/mL of the tested strain. 2 μL of each compound solution were spotted on the surface of the inoculated medium and incubated at 37 °C for 24 h. Controls included the vehicle (100% DMSO) or 2-µL spots of an antibiotic (ampicillin, chloramphenicol and ciprofloxacin) solution in sterile milliQ water (5 mg/mL, *i.e.* each spot contained 10 µg of the antibiotic). The results were recorded as the diameter of the growth inhibition zone.

MIC values of the compounds were determined using the broth microdilution method as recommended by Clinical Laboratory Standards Institute^76^. Bacterial inoculum was 5 × 10^4^ CFU/well. MICs were recorded after 18 h of incubation at 35 °C.

## Supplementary Material

Supplemental Material
