# Supplementary material for: Benzenesulfonamide derivatives as Vibrio cholerae carbonic anhydrases inhibitors: a computational-aided insight in the structural rigidity-activity relationships
Source: J Enzyme Inhib Med Chem. 2023 Apr 19;38(1):2201402. doi: 10.1080/14756366.2023.2201402 (PMC10120512; doi:10.1080/14756366.2023.2201402)
Supplement: Supplemental Material [file IENZ_A_2201402_SM3262.pdf]

SUPPORTING INFORMATION

for

Benzenesulfonamide derivatives as *Vibrio cholerae* carbonic anhydrases inhibitors: a computational-aided insight in the structural rigidity-activity relationships

Marialuigia Fantacuzzi[a,†], Ilaria D'Agostino[a,†], Simone Carradori[a,*], Francesco Liguori[a], Fabrizio Carta[b], Mariangela Agamennone[a], Andrea Angeli[b], Filomena Sannio[c], Jean-Denis Docquier[c,d], Clemente Capasso[e], Claudiu T. Supuran[b]

[a]Department of Pharmacy, "G. d'Annunzio" University of Chieti-Pescara, 66100 Chieti, Italy

[b]Neurofarba Department, Section of Pharmaceutical and Nutraceutical Sciences, University of Florence, 50019 Sesto Fiorentino, Florence, Italy

[c]Department of Medical Biotechnologies, University of Siena, Siena, Italy

[d]InBioS, Center for Protein Engineering, University of Liège, Liège, Belgium

[e]Department of Biology, Agriculture and Food Sciences, National Research Council (CNR), Institute of Biosciences and Bioresources, 80131 Naples, Italy

[†]These authors contribute equally

[*]Corresponding author: Simone Carradori PhD

Content

Figure S1. Ligand Root Mean Square Fluctuation (RMSF).

Figure S2. Protein-Ligand Root Mean Square Deviation (RMSD).

Figure S3. Predicted 3D binding mode and corresponding ligand interaction diagram of most selective ligands **12c**, **4b**, and **10a** in *h*CA I.

Figure S4. Predicted 3D binding mode and corresponding ligand interaction diagram of most selective ligands **12c**, **4b**, and **10a** in *h*CA II.

Figure S5. Predicted 3D binding mode and corresponding ligand interaction diagram of most selective ligands **12c**, **4b**, and **10a** in *Vch*βCA.

Figure S6. Predicted 3D binding mode and corresponding ligand interaction diagram of most selective ligands **12c**, **4b**, and **10a** in *Vch*γCA.

Table S1. Physico-chemical and pharmacokinetic properties of all the studied ligands.

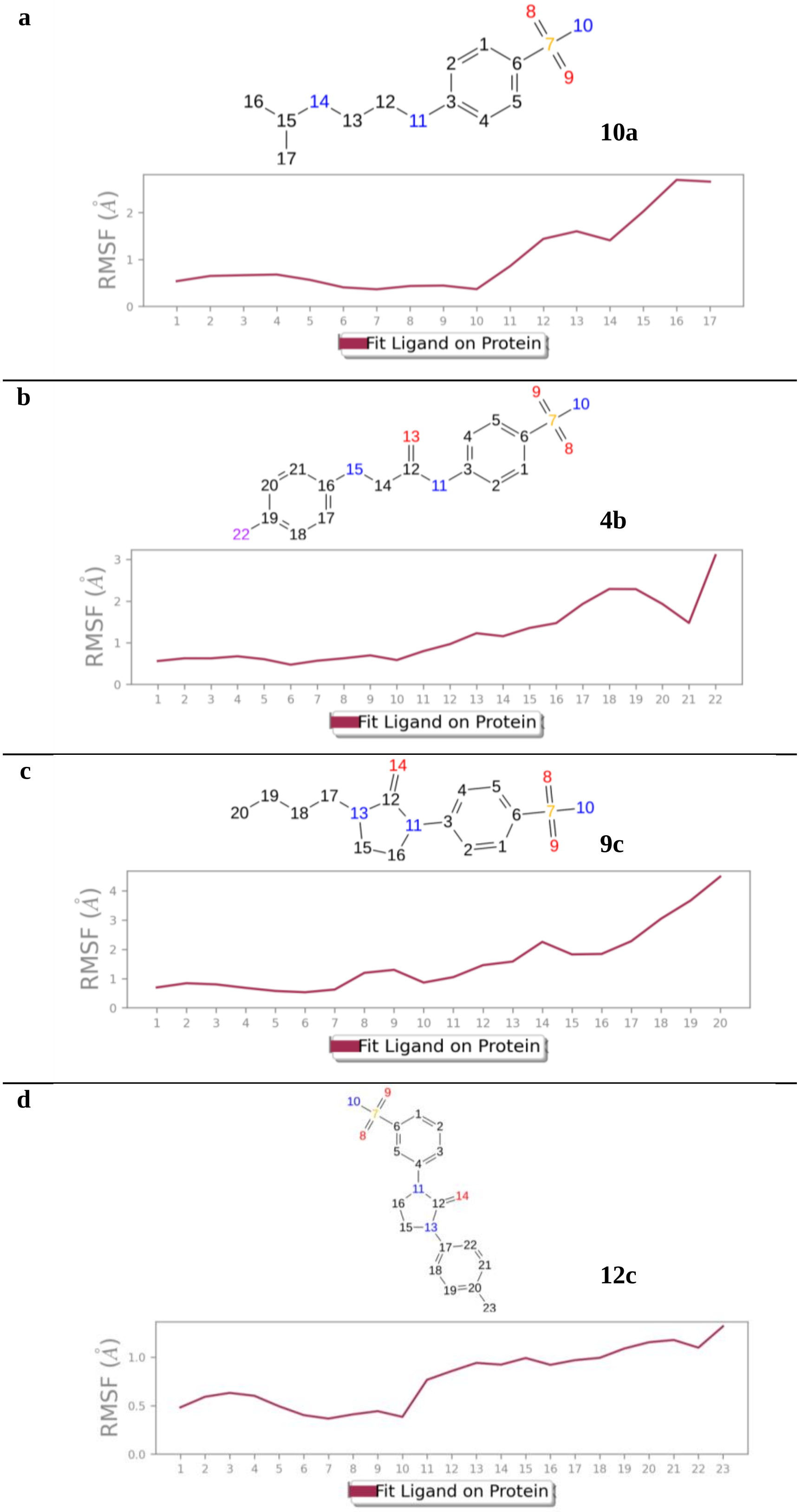

Figure S1. Ligand Root Mean Square Fluctuation (RMSF) for the ligands (**a**) **10a**, (**b**) **4b**, (**c**) **9c**, and (**d**) **12c** in *V*chαCA.

a

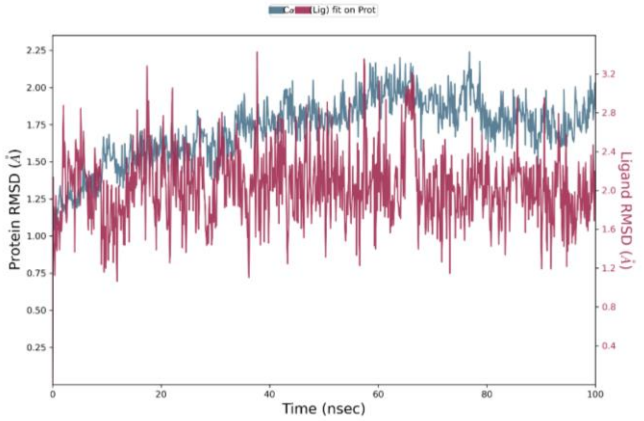

10a

b

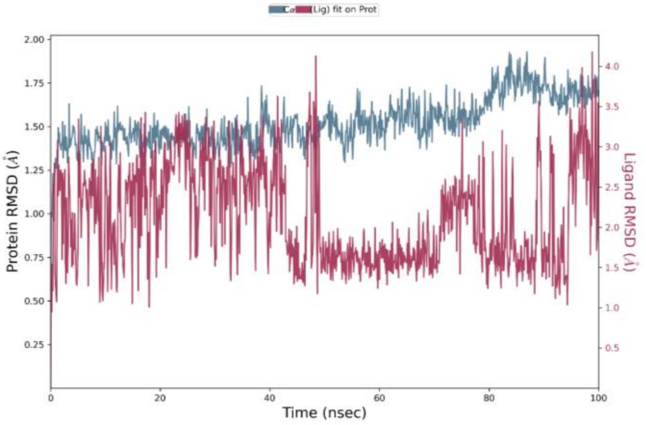

4b

c

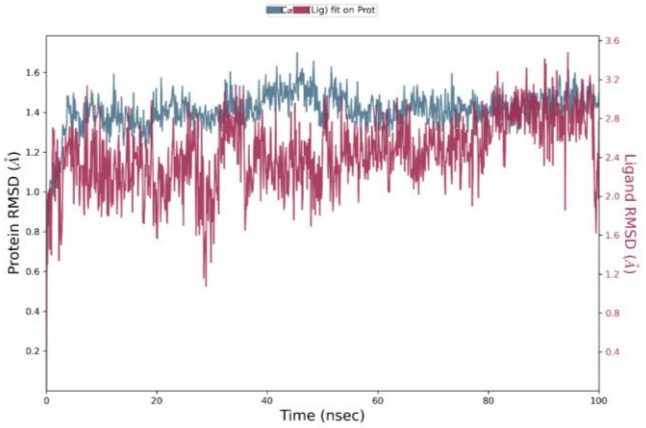

9c

d

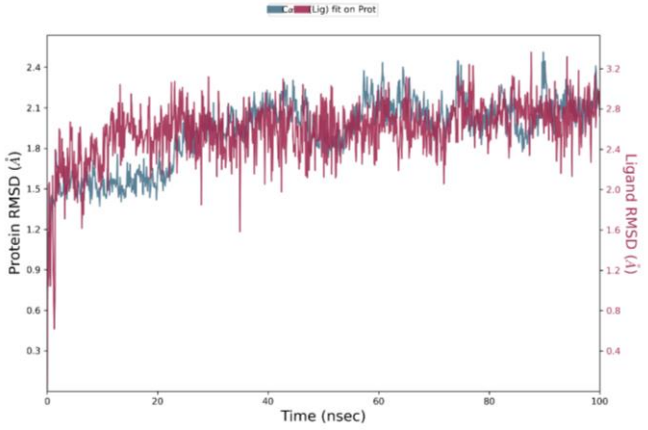

12c

Figure S2. Protein-Ligand Root Mean Square Deviation (RMSD). RMSD evolution during the MD simulation of the protein (light blue, calculated on the C-alpha, left Y-axis) and the ligands (**a**) **10a**, (**b**) **4b**, (**c**) **12c**, and (**d**) **9c** in *V*chαCA (calculated on all atoms, right Y-axis)

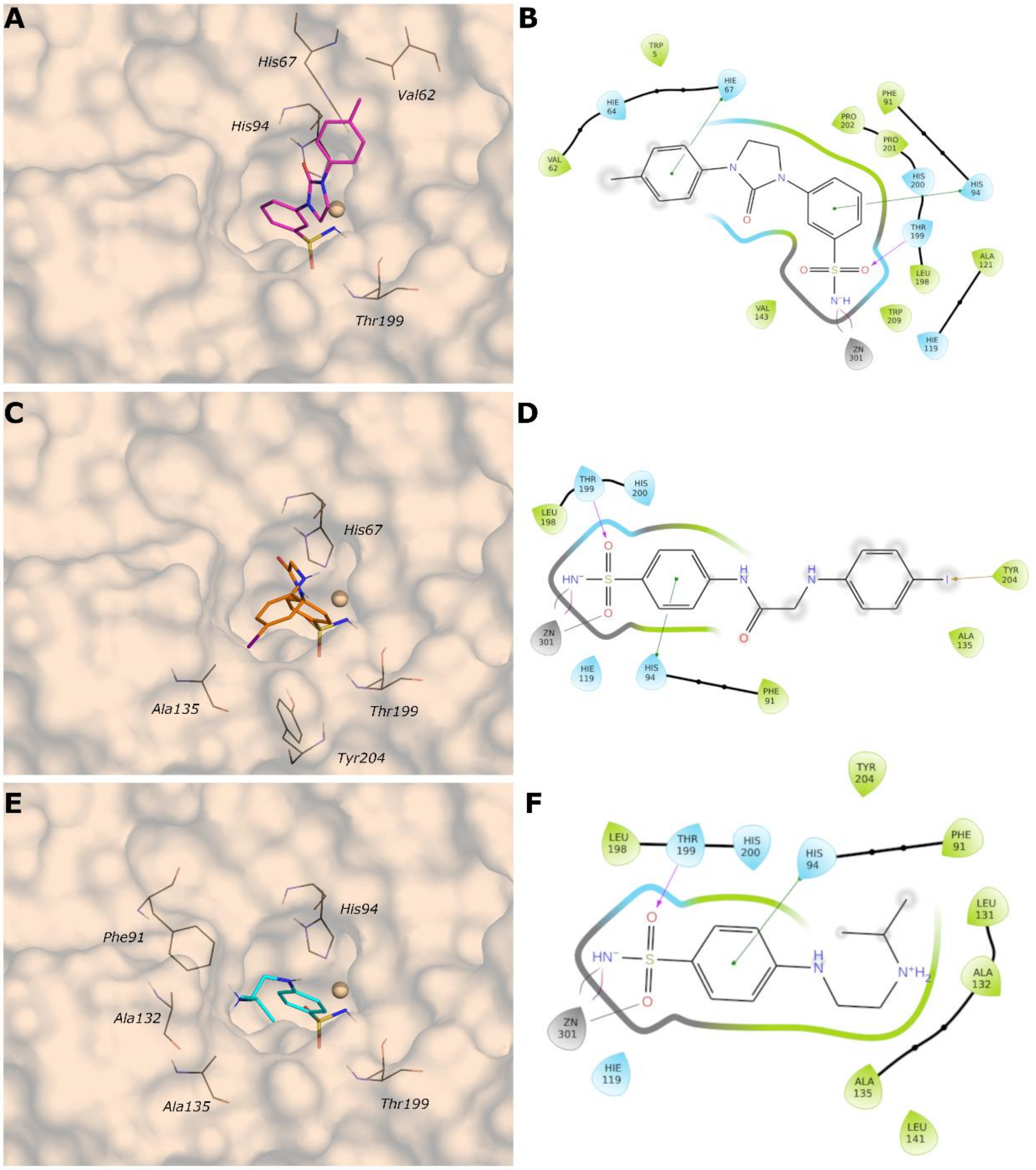

Figure S3. (**A**, **C**, and **E**) Predicted 3D binding mode and (**B**, **D**, and **F**) corresponding ligand interaction diagram of most selective ligands (**A-B**) **12c** magenta, (**C-D**) **4b** orange, and (**E-F**) **10a** cyan within *h*CA I (grey). The compounds are represented as sticks, and the protein surface is visualized.

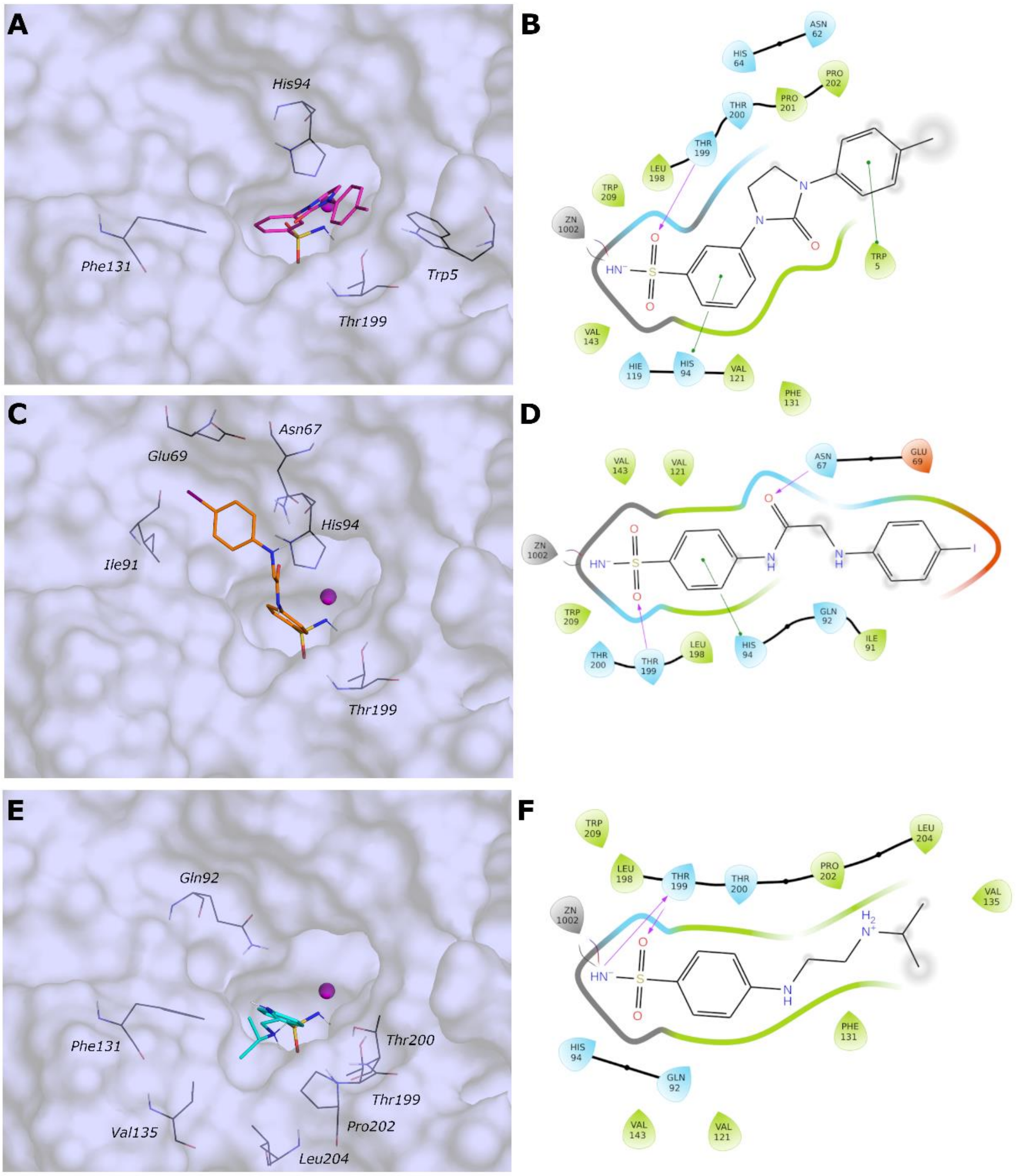

Figure S4. (**A**, **C**, and **E**) Predicted 3D binding mode and (**B**, **D**, and **F**) corresponding ligand interaction diagram of most selective ligands (**A-B**) **12c** magenta, (**C-D**) **4b** orange, and (**D-E**) **10a** cyan within *h*CA II (cyan). The compounds are represented as sticks, and the protein surface is visualized.

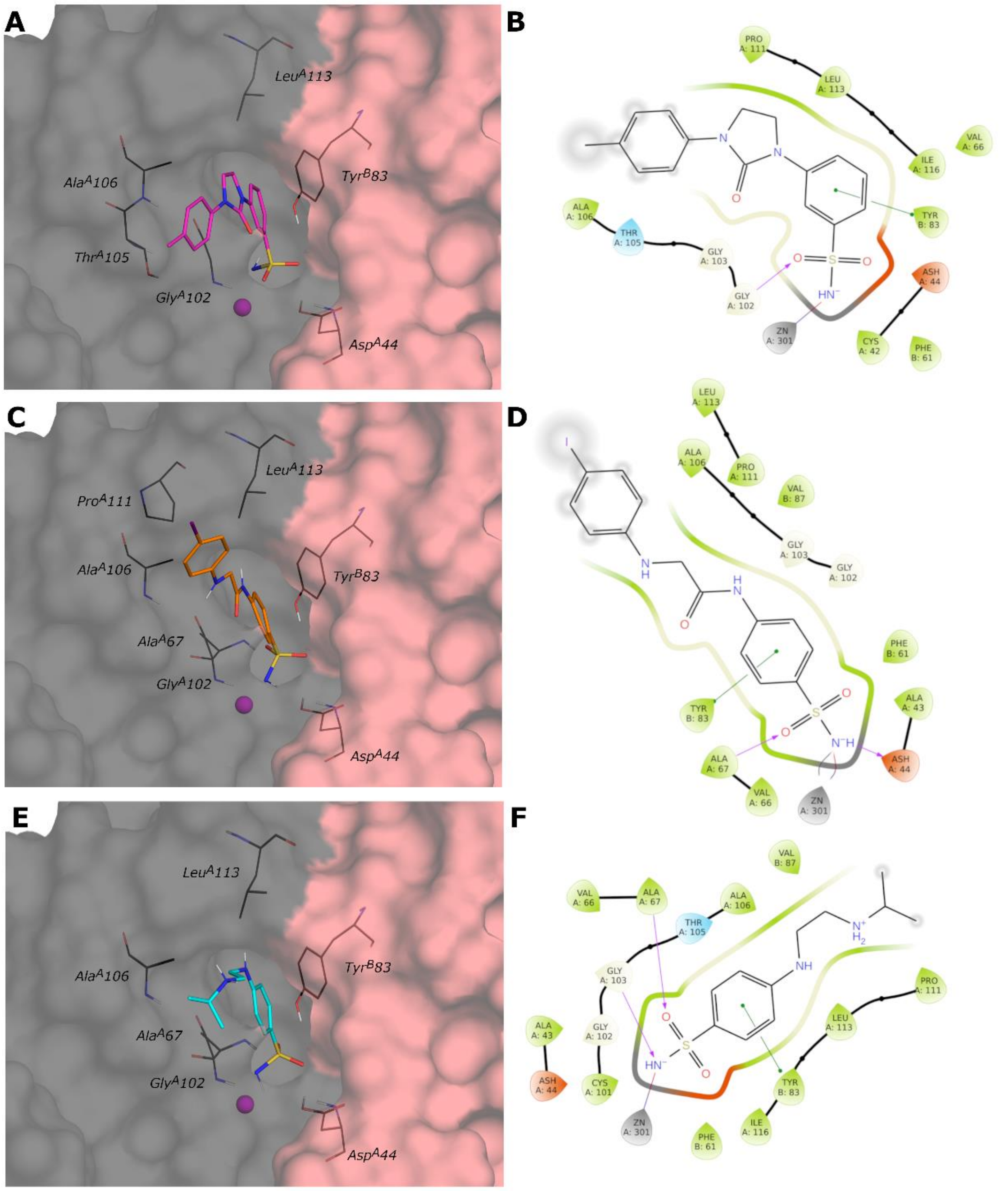

Figure S5. (**A**, **C**, and **E**) Predicted 3D binding mode and (**B**, **D**, and **F**) corresponding ligand interaction diagram of most selective ligands (**A-B**) **12c** magenta, (**C-D**) **4b** orange, and (**E-F**) **10a** cyan within *Vch*βCA (grey and pink). The compounds are represented as sticks, and the protein surface is visualized.

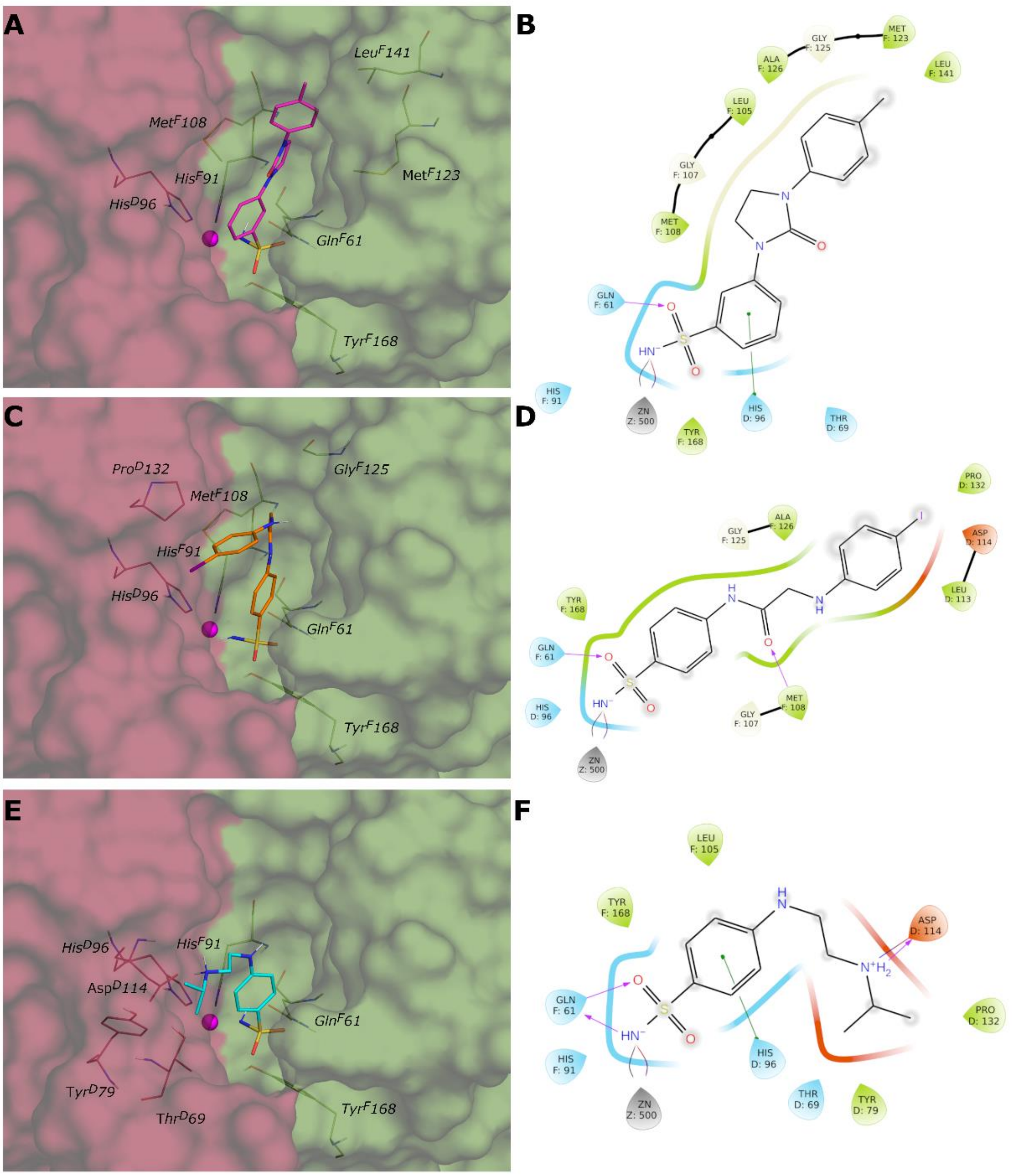

Figure S6. (**A**, **C**, and **E**) Predicted 3D binding mode and (**B**, **D**, and **F**) corresponding ligand interaction diagram of most selective ligands (**A-B**) **12c** magenta, (**C-D**) **4b** orange, and (**E-F**) **10a** cyan within *Vch*γCA (green and red). The compounds are represented as sticks, and the protein surface is visualized.

Table S1. Physicochemical and pharmacokinetic properties of the studied ligands.

cpd	MW	donor HB	accpt HB	QPlogPo/w	Rule Of Five	PSA	CIQP logS	*h* Oral Abs	% *h* Oral Abs	QPP Caco	QPP MDCK	QP logBB	QP logHERG	QP logKhsa
**1a**	291.367	4	6.5	1.076	0	89.142	-3.235	3	76.51	261.317	117.937	-1.52	-6.104	-0.419
**2a**	305.394	4	6.5	1.122	0	89.142	-3.483	3	76.775	261.299	117.928	-1.568	-6.023	-0.298
**3a**	325.812	4	6.5	1.477	0	89.133	-3.851	3	78.859	261.472	290.789	-1.381	-6.019	-0.331
**4a**	417.264	4	6.5	1.945	0	89.13	-5.579	3	81.602	261.531	338.347	-1.369	-6.081	-0.293
**5a**	370.263	4	6.5	1.476	0	88.453	-4.678	3	79.227	274.38	293.285	-1.35	-6.002	-0.313
**6a**	309.358	4	6.5	1.133	0	87.621	-3.554	3	78.541	325.084	254.115	-1.324	-5.984	-0.389
**7a**	305.394	4	7	0.964	0	90.698	-2.283	3	64.187	58.276	25.78	-1.261	-6.877	-0.397
**8a**	305.394	4	7	0.964	0	90.698	-2.283	3	64.187	58.277	25.78	-1.261	-6.877	-0.397
**9a**	271.377	4	7	0.372	0	90.281	-1.342	3	61.241	62.308	27.713	-1.305	-6.024	-0.551
**10a**	257.35	4	7	0.15	0	89.372	-1.114	3	60.538	67.277	30.11	-1.064	-5.688	-0.586
**11a**	291.367	4	6.5	1.172	0	89.717	-3.235	3	76.975	258.137	116.926	-1.528	-6.121	-0.416
**12a**	305.394	4	6.5	1.275	0	89.717	-3.483	3	77.577	258.12	116.917	-1.576	-6.039	-0.294
**13a**	325.812	4	6.5	1.299	0	89.705	-3.851	3	77.726	258.333	288.35	-1.389	-6.035	-0.328
**14a**	370.263	4	6.5	1.681	0	89.035	-4.678	3	80.325	270.792	290.325	-1.358	-6.016	-0.309
**15a**	271.377	4	7	0.312	0	90.917	-1.342	3	60.79	61.505	27.456	-1.311	-6.03	-0.548
**1b**	305.351	4	8	0.69	0	112.076	-3.028	3	68.745	128.71	54.834	-1.79	-6.133	-0.536
**2b**	319.378	4	8	0.941	0	112.442	-3.269	3	69.738	121.045	51.314	-1.894	-6.118	-0.426

cpd	MW	donor HB	accpt HB	QPlogPo/w	Rule Of Five	PSA	CIQP logS	h Oral Abs	% h Oral Abs	QPP Caco	QPP MDCK	QP logBB	QP logHERG	QP logKhsa
**3b**	339.796	4	8	1.188	0	112.069	-3.629	3	71.66	128.757	135.157	-1.658	-6.05	-0.457
**4b**	431.247	4	8	1.292	0	112.066	-5.326	3	72.272	128.769	157.239	-1.649	-6.109	-0.422
**5b**	384.247	4	8	1.196	0	111.679	-4.439	3	71.946	132.765	132.017	-1.647	-6.079	-0.439
**6b**	323.341	4	8	0.907	0	111.955	-3.338	3	70.073	129.721	93.779	-1.712	-6.086	-0.509
**7b**	320.365	3.25	7.75	1.162	0	124.207	-3.401	3	67.903	80.96	33.232	-2.09	-6.009	-0.385
**8b**	319.378	4	8.5	0.336	0	113.591	-2.066	2	55.594	30.956	13.002	-1.554	-7.131	-0.525
**9b**	285.36	4	8.5	-0.2	0	114.174	-1.14	2	51.871	28.723	11.996	-1.63	-6.108	-0.678
**10b**	271.334	4	8.5	-0.445	0	112.096	-0.917	2	52.237	36.19	15.399	-1.234	-5.534	-0.69
**11b**	305.351	4	8	0.694	0	112.841	-3.028	3	68.329	121.616	51.859	-1.835	-6.21	-0.537
**12b**	319.378	4	8	0.958	0	112.377	-3.269	3	70.312	128.698	55.123	-1.849	-6.063	-0.425
**13b**	339.796	4	8	1.142	0	112.823	-3.629	3	70.955	121.715	127.884	-1.703	-6.122	-0.457
**14b**	384.247	4	8	1.202	0	111.98	-4.439	3	72.068	134.213	134.318	-1.639	-6.072	-0.438
**15b**	285.36	4	8.5	-0.229	0	114.529	-1.14	2	51.71	28.746	12.071	-1.63	-6.113	-0.677
**1c**	317.362	2	6.5	1.644	0	93.508	-3.986	3	78.758	227.456	101.471	-1.203	-5.64	-0.094
**2c**	331.389	2	6.5	1.895	0	93.511	-4.254	3	80.322	230.319	102.852	-1.239	-5.534	0.038
**3c**	351.807	2	6.5	2.121	0	93.493	-4.648	3	81.556	227.611	250.256	-1.066	-5.571	0.004
**4c**	443.258	2	6.5	2.277	0	93.489	-6.457	3	82.468	227.672	291.205	-1.056	-5.641	0.047
**5c**	396.258	2	6.5	2.192	0	93.225	-5.52	3	82.702	250.12	237.88	-1.034	-5.581	0.033

cpd	MW	donor HB	accpt HB	QPlogPo/w	Rule Of Five	PSA	CIQP logS	h Oral Abs	% h Oral Abs	QPP Caco	QPP MDCK	QP logBB	QP logHERG	QP logKhsa
**6c**	335.352	2	6.5	1.864	0	93.467	-4.33	3	80.192	231.881	154.101	-1.123	-5.553	-0.048
**7c**	332.376	2	7.5	1.472	0	103.244	-3.913	3	77.121	209.839	93.003	-1.296	-5.549	-0.14
**8c**	331.389	2	6.5	1.747	0	93.04	-4.254	3	78.726	209.73	92.951	-1.305	-5.315	-0.031
**9c**	297.371	2	6.5	1.455	0	95.123	-3.249	3	76.996	209.196	92.695	-1.454	-4.882	-0.205
**10c**	283.345	2	6.5	1.067	0	94.497	-2.989	3	75.014	216.997	96.438	-1.201	-4.394	-0.262
**11c**	317.362	2	6.5	1.63	0	93.578	-3.986	3	78.721	228.856	102.712	-1.19	-5.596	-0.1
**12c**	331.389	2	6.5	1.888	0	93.581	-4.254	3	80.227	228.807	102.689	-1.242	-5.54	0.035
**13c**	351.807	2	6.5	2.106	0	93.563	-4.648	3	81.511	228.965	253.266	-1.054	-5.529	-0.002
**14c**	396.258	2	6.5	2.2	0	93.301	-5.52	3	83.256	266.946	256.562	-0.992	-5.528	0.028
**15c**	297.371	2	6.5	1.435	0	94.962	-3.249	3	77.077	214.524	95.767	-1.429	-4.826	-0.214
**AAZ**	222.236	3	9	-1.768	0	133.243	-1.021	2	44.375	35.654	24.104	-1.802	-3.791	-0.993

MW: molecular weight; donorHB: H-bond donor; accept: H-bond acceptor; QPlogPo/w: Predicted octanol/water partition coefficient (-2.0 – 6.5); RuleOfFive: Number of violations of Lipinski rule of five; PSA: Van der Waals surface area of polar nitrogen and oxygen atoms and carbonyl carbon atoms (7 – 200); CIQPlogS: Conformation Independent predicted aqueous solubility, log S. S in mol dm^{-3} is the concentration of the solute in a saturated solution (-6.5 – 0.5); *h* Oral Abs: human oral absorption (1, 2, or 3 for low, medium, or high); % *h* OralAbs: Predicted human oral absorption on a 0 to 100% scale; QPPCaco: Predicted apparent Caco-2 cell permeability in nm/sec. Caco-2 cells are a model for the gut–blood barrier (500 great); QPPMDCK: Predicted apparent MDCK cell permeability in nm/sec. MDCK cells are considered to be a good mimic for the blood–brain barrier (500 great); QPlogBB: Predicted brain/blood partition coefficient (-3.0 – 1.2); QPlogHERG: Predicted IC_{50} value for the blockage of HERG K^+ channels (concern below -5); QPlogKhsa: Prediction of binding to human serum albumin (-1.5 – 1.5).
